# Postbiotics: an insightful review of the latest category in functional biotics

**DOI:** 10.1007/s11274-025-04483-8

**Published:** 2025-08-02

**Authors:** Ayodeji Amobonye, Brynita Pillay, Felicity Hlope, Stella Tofac Asong, Santhosh Pillai

**Affiliations:** 1 Department of Biotechnology and Food Science, Faculty of Applied Sciences, Durban University of Technology, P. O. Box 1334, Durban, 4000 South Africa; 2 Department of Polymer Chemistry and Technology, Kaunas University of Technology, Radvilenu Rd. 19, 50254 Kaunas, Lithuania

**Keywords:** Functional biotics, Nutraceuticals, Parabiotics, Postbiotics, Prebiotics

## Abstract

Postbiotics have recently emerged as one of the latest functional food products due to the ever-evolving landscape for microbiome-targeted health interventions. Postbiotics, along with other functional biotics, viz., probiotics, prebiotics and synbiotics, confer their health benefits mainly via the modulation of the gut microbiota. Postbiotics are considered more promising than probiotics as they elicit similar effects, despite being inactivated, thus relegating concerns of strain activity and stability that have been raised about probiotics. This review attempts to provide critical insights into postbiotics by firstly revising its definition to create a streamlined framework for further discourse on the interplay between postbiotics, nutrition, microbiota, and health. Similarly, this review establishes the nexus between postbiotics and probiotics while highlighting that postbiotics can also be derived from other microbes apart from lactobacilli such as yeasts and fungi. Furthermore, an overview of the extraction and production of postbiotics are presented as well as the biochemistry of short-chain fatty acids, enzymes, peptides, polysaccharides, peptidoglycans and teichoic acids, which have all been identified as postbiotic components. Finally, their bioactivities (antioxidant, anti-inflammatory, antidiabetic, immunomodulatory, anti-hypertensive, antimicrobial) and the patent landscape of postbiotics are evaluated to promote its innovative applications in the food, veterinary, pharmaceutical and cosmetic industries. Having identified major gaps and areas of improvement, it is believed that this critical review will serve as a guide in the increasing effort to advance the industrial potential of postbiotics.

## Introduction: a paradox of nomenclature

The age-long consumption of fermented foods by various cultures across the world has been associated with various health benefits, especially the attenuation of intestinal disturbances, which have been validated by various scientific investigations (Mukherjee et al. 2024). It is believed that the relationship between traditional fermented foods, their natural microbiome and their biological activities was first developed into the scientific concept of probiotics in 1907 by Elie Metchnikoffa (Russian scientist), after he had observed the consumption of sour dairy products containing beneficial microbes was related to the longevity and good health of Bulgarian peasants (Krawczyk And Banaszkiewicz 2021). The science of functional biotics which was initially centred around the health-beneficial effects accruable from viable cells, viz., probiotics, has now expanded to include the various derivatives of the cells such as their metabolites, the inactivated (dead) cells as well as substrates which promote the growth of such beneficial microbes. Thus, having been established that various health benefits can be obtained beyond the viability of probiotic microbes, other functional biotic classes, including postbiotics, prebiotics, and synbiotics, have now attracted the attention of scientists, consumers, the industry and other relevant stakeholders.

Postbiotics (sometimes referred to as metabiotics, biogenics), in particular, have become very prominent in both nutrition and health sciences as they are believed to confer various health benefits to the consumers/patients without the attendant concerns arising from the ingestion of probiotics in their living forms. However, like many other emerging concepts, serious debates exist about the definition of postbiotics as the critical review of both primary and secondary research papers reveals a lack of total consensus. Although some common denominators exist in some current definitions, the situation where various cellular derivatives are termed postbiotics presents a wide knowledge gap, making comparison between various findings non-feasible. For instance, various studies consider cell-free supernatants derived from the fermentation of probiotics as postbiotics (Dameshghian et al. 2024; Kim et al. 2024a, SAntana et al. 2024), while in some other works heat-killed probiotic cells devoid of the supernatants (Kim et al. 2024b). Furthermore, some authors considered the inactivated probiotic cells processed together with the supernatants as postbiotics (Kim et al. 2023; Xu et al. 2023a, b).

However, in this review, the widely accepted definition by the International Scientific Association of Probiotics and Prebiotics (ISAPP) which states that “postbiotics is a preparation of inanimate microorganisms and/or their components that confers a health benefit on the host”, will be considered as the standard. In adopting the ISAPP definition, this review does not in any way invalidate the other definitions but appreciates the fact that the matter of nomenclature is an open and ongoing debate, which should not be a major stumbling block to the current advancements being made in this area of research. The authors also believe that baring the “invention” of a globally acceptable definition by international regulatory bodies such as the WHO, FAO or Codex Alimentarius, the opinion of ISAPP- which is a consortium of scientific experts focused on advancing the science of probiotics, prebiotics and related substances- would be less subjective than those held by various independent laboratories around the world. Furthermore, considering that “postbiotics” is still a relatively emerging concept, it is expedient to have a more inclusive definition which considers postbiotics as the encompassing term for heat-killed probiotics, heat-inactivated probiotics, ghost-probiotics, non-viable probiotics, paraprobiotics, cell fragments, cell lysates and cell-free supernatants.

As earlier stated, the key highlight of postbiotics is they confer health-promoting effects despite their “non-living” status, thus allaying fears of any detrimental effect on immunocompromised consumers (Sabahi et al. 2023; Sadeghi et al. 2023). Compared to probiotics, the use of postbiotics also minimises the potential risk of antibiotic resistance, and they are more stable during processing as well as during storage (Sabahi et al. 2023). The health benefits of postbiotics have been posited to be pleiotropic and these benefits are elicited via direct interaction with the host’s system, the host’s gut microbiome or a combination of both (Uhlig et al. 2023). Furthermore, the various bioactivities attributed to postbiotics are majorly a function of their components, which include bacteriocins, cell wall components, enzymes, exopolysaccharides, organic acids, peptidoglycan, short-chain fatty acids (SCFA) and other soluble bioactive compounds (Mehta et al. 2023; Sadeghi et al. 2023). Consequently, bioactivities due to living cells or any of the metabolite components in the purified form cannot be regarded as due to the activity of the postbiotic. Furthermore, these components synergistically elicit various local benefits, including anti-inflammatory, antimicrobial and immunomodulation, as well as systemic effects such as anti-obesogenic, anti-proliferative, antihypertensive, hypercholesterolaemic on the host physiological functions (Aguilar-Toalá et al. 2018). In this regard, various experimental and clinical studies have demonstrated the ability of postbiotic preparations to ameliorate various disease conditions or modulate homeostatic balance in humans (Fang et al. 2023; Rui et al. 2024) and other animals such as poultry birds (Johnson et al. 2019; Chaney et al. 2023), cattle (Ríus et al. 2022; Dai et al. 2024).

Contrary to the general notion that postbiotic preparations are typically derived from probiotic bacteria, especially from the *Lactobacilli* and *Bifidobacterium* genera, health-promoting postbiotics can also be produced from yeasts (Xu, et al. 2023a; Dai et al. 2024), and filamentous fungi (Jasim et al. 2022) that may not be systemically classified as probiotics. Consequently, drawing from their many benefits and versatility, various post-biotic-based products are currently being marketed worldwide with remarkable success. These products include postbiotic supplements for humans and animals, human cosmetics, as well as key ingredients in the food/beverage formulation and packaging. According to data from Allied Market Research (2021), the global postbiotic market was estimated at USD 1.6 billion in 2021 and was projected to grow at a CAGR of 6.8% to USD 3 billion in the next decade.

Despite being an emerging field, many review articles have been written on postbiotics, although mostly focusing on their production, processing, and health benefits. However, some of these articles are limited in scope, especially due to their unambiguous definition of the term ‘postbiotics’. Furthermore, the industrial applicability and the economic potential of this biotic preparation have received little or no attention. Therefore, having addressed the nomenclature paradox, the other objectives of this paper are to address the aforementioned gaps by critically analysing recent literature on the concept of postbiotics with special emphasis on the wide spectrum of source microbes, processing, biochemical composition and their biological activities. In addition, the paper also evaluates the current and potential industrial uses of postbiotics, their commercial potential as well as a brief overview of the patents space. Finally, the pertinent challenges and limitations encumbering the postbiotic concept are also identified, while possible solutions and future perspectives were also put forward.

### Probiotics to postbiotics

According to ISAPP, probiotics are “live organisms that, when administered in adequate amounts, confer a health benefit on the host”. These beneficial microbes are mainly obtained from fermented foods, as well as the gut microbiota of fishes (aquaculture), humans and animals, which all contain a plethora of strain-specific probiotics (Cai et al. 2019). Probiotics are mostly lactic acid bacteria (LAB) which include members of the *Lactobacillus* (Zhang et al. 2020), *Lactococcus* (Saeed et al. 2023) *Leuconostoc* (Alan et al. 2022), and *Bifidobacterium* (Alan et al. 2022) genera. However, eukaryotic organisms such as yeasts and fungi, have also been demonstrated to serve as probiotics, especially the yeast probiotics *Saccharomyces cerevisiae* and *S. boulardii* which produce a wide range of health-benefiting metabolites (Fu et al. 2023). Recently, the potential of some filamentous fungi as probiotics was also highlighted; for instance, *Aspergillus niger* served as a growth-promoting component in aquaculture feeds (Jasim et al. 2022). The benefits of probiotics include protection against pathogens (Popova et al. 2012), immunomodulation to aid in the regulation of the host's immune responses (Azad et al. 2018), alleviating lactose intolerance (Gingold-Belfer et al. 2020), prevention of dental caries (Shi et al. 2023), adjuvants in hypercholesterolemia, cancer and hypertension (Latif et al. 2023), and maintenance of the intestinal microbiota and intestinal barrier function (Liu et al. 2024).

Recent advancements in understanding the dynamics and mechanisms of probiotics have led to a progressive transition in the use of live probiotic cultures, shifting towards their non-living metabolic byproducts, commonly known as postbiotics. It has since been established that microbial viability may not be necessary to attain the derived health benefits from functional biotics. In this context, Zhang et al. (2022) compared the effects of the probiotic, *Bifidobacterium adolescentis* B8589, and the postbiotics derived from it and observed that both were capable of improving disease phenotypes, thus highlighting postbiotics as next-generation biotherapeutics. The shift to postbiotics has continued to rise following concerns about the potential health dangers of consuming probiotics, which are live organisms. Some of these dangers include antibiotic resistance genes (ARGs), limited storage potential due to the number of viable cells being reduced throughout the product shelf life (Tingirikari et al. 2024), and the potential to negatively influence the balance between anti and pro-inflammatory cytokines amongst at-risk groups such as immunocompromised individuals, patients that have abnormal gastrointestinal barriers, patients following surgical treatment and premature newborns (Siciliano et al. 2021; Tingirikari et al. 2024). Although many probiotics have been conferred with the GRAS (generally recognised as safe) status, instances of horizontal transfer of antibiotic resistance genes (ARGs) have been recorded. For example, antimicrobial resistance (AMR) gene transfer was reported by Baumgardner et al. (2021) from commercial animal probiotics using PCR screening for known AMR genes such as tetM, ermB, sul1, and dfrG, to name a few; more than 90% of the 47 probiotics contained at least one AMR gene, thus heightening the probability of AMR gene transmission to gastrointestinal microbiota. Similarly, spore-forming *Bacillus* spp. collected from 50 probiotic products displayed resistance to lincomycin and tetracycline (Deng et al. 2021). An additional study by Tian et al. (2024) found that streptomycin resistance from *Lactobacillus* probiotics was highly transferrable to the representative bacteria *Enterococcus faecalis, Staphylococcus aureus* and *E. coli* when co-incubated on human intestinal cells Caco-2, indicating its potential of elevating the rate of ARG transmission in the gut reservoir. A more serious case highlighting the adverse effects of probiotics was recently reported by Eze et al. (2024), where a 79-year-old patient with a history of chronic probiotic use, resulting in a resistant case of *Lactobacillus* bacteremia with no benefit from antibiotic therapy, ultimately leading to death due to multiple complications related the *Lactobacillus* bacteremia. Furthermore, postbiotics are also promoted by their remarkable stability during processing and storage, which has been a key challenge with live probiotics, enhancing their utilisation in regions with unreliable cold chains that may rely on ambient storage temperatures (Salminen et al. 2021). This desirable characteristic was highlighted by Arrioja-Bretón et al. (2020) who evaluated the stability of postbiotics derived from LAB under various storage conditions (15 °C, 25 °C and 35 °C). The findings showcased that the highest bioactivity was maintained when stored at 15—25 °C, thus emphasising the suitability of ambient temperatures for preserving postbiotic functions.

In addition, postbiotic preparations are recovered as extracellular secretions during the fermentation of probiotics; hence, from a functional perspective, the processing of postbiotics is less demanding (Blazheva et al. 2022). Besides their ease of processability and stability, the safety dose limits and unique chemical structures of postbiotics also enhance their applications as functional biotic products for human and animal use (Mohammed and Çon 2024). Consequently, a wide range of postbiotics have been prepared from bacteria, yeasts as well as filamentous fungi (Table 1). It is not unexpected that most of the postbiotics were derived from *Lactobacillus* and *Bifidobacterium* genera, which have been well described for their tendencies to mainly produce beneficial organic acids and short-chain fatty acids, respectively, during fermentation. However, various postbiotics have also been prepared using bacteria from other genera, such as *Bacillus*, *Enterococcus*, and *Streptococcus* strains (Table 1). Although the potentials of various yeast species, such as *Debaryomyces hansenii* (Angulo et al. 2020), *Kluyveromyces marxianus* (Nag et al. 2023), and *Pichia kudriavzevii* (Zhang et al. 2023), to produce postbiotics have been demonstrated, much focus has been placed on detailing the composition and biological activities of *Saccharomyces* derived postbiotics. On the other hand, the production and characterisation of postbiotics from *Aspergillus*, *Penicillium,* and mushrooms such as *Cordyceps* and *Phellinus* species have also been demonstrated (Seidler et al. 2024).

**Table 1:** Postbiotics derived from various probiotic microorganisms

Probiotic	Postbiotic component(s)	Reference
Bacteria		
*Bacillus amyloliquefaciens* J	Phenyllactic acid, lactic acid, acetic acid, butyric acid, propionic acid, pentanoic acid, phenolic acid, ferulic acid	Tong et al. 2023
*Bacillus coagulans* GBI-30	Myristic/capric/palmitic/stearic acids, cysteine, tryptophan, tyrosine, alanine, phenylalanine, threonine and flavin adenine dinucleotide	Aguilar-Toalá et al. 2020
*Bacillus velezensis* Kh2-2	Acetic acid, propionic acid, isobutyric acid, isovaleric acid, valeric acid	Chen et al. 2023
*Bacillus velezensis* KMU01	Phenylalanine, tyrosine, tryptophan, valine, leucine, isoleucine, GABA, glutamine, cystine, histidine, proline, arginine	Jung et al. 2022; Chen et al. 2023
*Bifidobacterium longum* CECT-7347	Whole cell postbiotic	Martorell et al. 2021
*Bifidobacterium breve* BB091109 (NCIMB43992)	β-glucans	Motei et al. 2023
*Bifidobacterium lactis* BB12	Linoleic acid, exopolysaccharides (glucose, galactose, glucuronic acid, rhamnose and xylose), bacteriocins (26, 45 and 95 kDa)	Amiri et al. 2021
*Enterococcus faecium* EFM2	Glucose, 5-bromo-4-chloro-3-indoxyl-β–glucoside, β-glucopyranoside indoxyl, phenylalanine arylamidase, tyrosine arylamidase, galactose, cellobiose, maltose, -mannosidase, maltotriose, esculin hydrolysis, L-arabinose, leucine arylamidase, pyrrolydonyl-arylamidase, arginine	Kim et al. 2024a
*Enterococcus faecium* NCIM 5593	γ-aminobutyric acid (GABA)	Gangaraju et al. 2022
*Enterococcus faecium* EK13	Enterocins (EntA(P)/EK13, EntM/AL41, Ent4231, Ent7420, Ent55, Ent9296, Ent412 and DurED26E/7)	Zábolyová et al. 2024
*Lacticaseibacillus casei* CRL 431	Myristic/capric/palmitic/stearic acids, cysteine, tryptophan, tyrosine, alanine, phenylalanine, threonine and flavin adenine dinucleotide	Aguilar-Toalá et al. 2020
*Lactobacillus helveticus* H9	Lactate. acetate, succinate, leucine, isoleucine, lysine, valine, phenylalanine, threonine, methionine, histidine, tryptophan, glutamic acid, glutamine, proline, asparagine, aspartic acid, serine, tyrosine, alanine, arginine, glycine, cysteine	Rozhkova et al. 2023
*Lactiplantibacillus plantarum* KM1	Phenylalanine, tyrosine, tryptophan, valine, leucine, isoleucine, GABA, glutamine, cystine, histidine, proline, arginine	Jung et al. 2022
*Lacticaseibacillus paracasei* ABK	Lactate. acetate, succinate, leucine, isoleucine, lysine, valine, phenylalanine, threonine, methionine, histidine, tryptophan, glutamic acid, glutamine, proline, asparagine, aspartic acid, serine, tyrosine, alanine, arginine, glycine, cysteine	Rozhkova et al. 2023
*Lactiplantibacillus plantarum* SN4	Phenyllactic acid, lactic acid, acetic acid, butyric acid, propionic acid, pentanoic acid, phenolic acid, ferulic acid	Tong et al. 2023
*Letillactobacillus kefiri LK1*	Glucose, 5-bromo-4-chloro-3-indoxyl-beta-glucoside, β-galactopyranoside indoxyl, phenylalanine arylamidase, tyrosine arylamidase, 5-bromo-4-chloro-3-indoxyl-alpha-galactoside, arabinose, arbutin, L-proline arylamidase, Ala-Phe-Pro-arylamidase, phenylphosphonate	Kim et al. 2024a
*Lactococcus chungangensis* CAU 1447	Lactic, acetic acid, aspartic acid, glutamic acid, asparagine, serine, glutamine, histidine, glycine, alanine, GABA, valine, methionine, tryptophan, phenylalanine, isoleucine, lysine, proline, caproic acid, lauric acid, myristic acid, palmitoleic acid, stearic acid, oleic acid, linoleic acid	Nam et al. 2021
*Lactococcus lactis* subsp. *cremoris* WA2-67	Garavicin A, garavicin Q, nisin Z, nisin A	Feito et al. 2023
*Leuconostoc mesenteroides* (A4X, Z36P, B12, and O9)	Dextran, mannitol, oligosaccharides, riboflavin (Vitamin B2)	Zarour et al. 2024
*Leuconostoc pseudomesenteroides* (Y4, Y6, Y20, Y49 and Y51)	Maleic acid, citric acid, tartaric acid, pyruvic acid, malic acid, succinic acid, fumaric acid, acetic acid, acetoin, 2,3-butanediol	Ala n et al. 2022
*Leuconostoc mesenteroides* J.27	Lactic acid, acetic acid, citric acid, boric acid, succinic acid, methyl succinic acid, aminomalonic acid, glyceric acid, valine, alanine, lysine, glycine, tyramine, threonine, glucose, altrose, xylitol, sucrose, phosphoric acid, glycerol-3-phosphate, myo-inositol	Toushik et al. 2022
*Pediococcus acidilactici* (B-LC-20)	Gallic acid, protocaucic acid, procyanidin B2, catechin, 4-hydroxy benzoic acid, syringic acid, epicatechin, caftaric acid, chlorogenic acid, 2.5, di-hydroxy benzoic acid, p-coumaric acid, ferulic acid, rutin, quercetin-3-glucoside, kaempferol-3-glucoside, myricetin, quercetin, luteolin, kaempferol, propiolic acid, formic acid, acetic acid, propanoic acid, 2-methyl-propanoic acid, butanoic acid, 3-methyl-butanoic acid	İnci Li et al. 2021
*Pediococcus acidilactici* (B-LC-20, Bactoferm)	Linolenic acid, 2-propynoic acid, acetic acid, propanoic acid, 2-methyl propanoic acid, butanoic acid, 2-methyl butanoic acid, pentanoic acid, lactic acid, octanoic acid, decanoic acid, 3-phenyl-2-propenoic acid, alpha-hydroxy-benzenepropanoic acid, dodecanoic acid, ethyl propanoate, 2-hydroxy-methyl propanoate, ethyl butanoate, butyl acetate, ethyl pentanoate, ethanol, resveratrol, kaempferol-3-glucoside	İnci Li et al. 2021
*Streptococcus alactolyticus* FGM	Fumaric acid, pyruvic acid, linoleic acid, phenyl lactic acid, tartaric acid, xylose, lactic acid, homovanillic acid, indole lactic acid, glyceraldehyde, maleic acid, hydroxy phenyl lactic acid, phenyl pyruvic acid, ketoleucine, 3-methyl-2-oxopentanoic acid, fructose, glutaconic acid, *N*-acetyltryptophan, proline, creatine, glutamylalanine, glutamic acid, tryptophan, asparagine, glucaric acid, citramalic acid, aminocaproic acid, phenylalanine, glucose, ribulose, *N*-acetyaspartic acid, 2-methylbutyroylcarnitine, butyrylcarnitine, *N*-acetyalanine, suberic acid, methyl cysteine, glycyl proline, 3-hydroxybutyric acid, dimethylglycine, oxoadipic acid, 4-hydroxybenzoic acid, isocitric acid, malonic acid	Gu et al. 2024
*Streptococcus salivarius* K12	Salivabactin A, salivabactin B	Do et al. 2024
**Yeasts**		
*Saccharomyces bourlardii*	N-acryloylglycine, N6-acetyl-L-lysine, N-acetylhistidine, N-methylalanine, p-aminobenzoic acid, tyrosol, glycerophosphocholine, 1-aminocyclopropanecarboxylic acid, 2-hydroxy-4-methylpentanoic acid, shikimic acid, nicotinic acid, indoleacetaldehyde, serotonin, indolepyruvate, L-3-phenyllactic acid	Fu et al. 2023
*Saccharomyces cerevisiae* var*. boulardii* NCYC 3264	Succinic acid, D-glucose, 1,3-dioxolane, phenyl ethyl alcohol, amphetamine, 2-pentanoic acid, benzeneacetic acid, M-cymene, hydroxycinnamic acid, L-leucylglycine, DL-aspartic acid, amphetamine, citric acid, cinnamic acid, L-alanine, acetic acid, 2,4-dinitro-1-napthalenol, quinoline, erythromycin, vanillic acid, L-phenylalanine, vitamin B6, hydroxylamine, L-phenylisoquinoline, L-proline, D-proline, pyruvic acid, 2,4-bis(1,1-dimethylethyl)-phenol, fumaric acid, malic acid	Datta et al. 2017
*Saccharomyces cerevisiae* BY4742	D-glucose, citric acid, lactic acid, succinic acid, glycerol, fumaric acid, malic acid, valine, ethanolamine, isoleucine/leucine, glycerol, glycine, uracil, serine, threonine, homoserine, erythritol, cytosine, aspartate, A-ketoglutaric acid, glutamate, phenylalanine, ribose, asparagine, 2-aminoadipic acid, orotic acid, glycerol-1-phosphate, glutamine, N-acetyl-glutamic acid, ornithine, lysine, mannitol, tyrosine, inositol, xylulose-5-phosphate, glucose-6-phosphate	Datta et al. 2017
*Saccharomyces cerevisiae* KU200270, KU200280	β-glucan, GSH (glutathione), GSSG (glutathione disulfide), L-ornithine, spermidine, esterase, esterase lipase, leucine arylamidase, valine arylamidase, cystine arylamidase, trypsin, acid phosphatase, naphthol-AS-BI-phosphohydrolase, α-galactosidase, β-galactosidase, β-glucuronidase, α-glucosidase, β-glucosidase, N-acetyl-β-glucosaminidase, α-mannosidase, α-fucosidase	Lee, et al. 2019a
**Fungi**		
*Aspergillus flavus*	2,4-hexadienoic acid, methyl ester, 1,4-diphenylbut-3-ene-2-ol, 2-thiopheneethanol,5-(4,5-dihydro-4,4-dimethyl-2-oxazolyl), phenol, 2,4-Bis(1,1-dimethylethyl), caryophylla-4(2),8(13)-dien-5-ol,4 h-1-benzopyran-4-one,2-(3,4-dimethoxyphenyl)−3,5-dihydroxy-7-methoxy-,androstane-11,17-dione,3-[(trimethylesilyl)oxy]−17-[o-(phenylmethyl)oxime],(3a,5a), Tricyclo[4,3,1,1(3,8)]undecane-3-carboxylic acid, Silane, trimethyl (stigmast-5-en-3beta-yloxy)-, Cholestan-3-ONE,Cyclic 1,2-Ethanediyl Aetal,*(5 a)-, Curan, 16,17-didehydro-,(20.xi.)-, Bis(2-ethylhexyl)phthalate, Cyclohexane, hexaethyllidene-(isomer 1)	Alm Anaa et al. 2021
*Cordyceps militaris*	Uronic acid, mannose, glucose, galactose, α-glucan, β-glucan,	Kim et al. 2023
*Penicillin flavigenum*	Sorbicillin-like metabolites	Suh et al. 2023
*Phellinus linteus*	Uronic acid, rhamnose, fucose, arabinose, xylose, mannose, galactose, glucuronic acid, galacturonic acid, glucose, rhamnopyranosyl, fucopyranosyl, arabinofuranosyl, xylopyranosyl, galactopyranosyl, galactopyranosidic	Suh et al. 2023

### Extraction and processing of postbiotics

The extraction and processing of postbiotics involves various techniques which may be used individually or in combination, depending on the applicatory needs, as well as available resources (Table 2). The first step in the production of these biological preparations is the recovery of the fermentation broth (Tong et al. 2023). Prior to that, various fermentation conditions must be optimised for the probiotic culture to produce postbiotics both efficiently and effectively. For instance, the choice of substrate has been identified as a critical factor; it is believed that the typical use of commercial media, especially de Man, Rogosa & Sharpe (MRS) broth, is quite expensive and non-feasible for upscaling. Interestingly, various alternative substrates, such as xylooligosaccharides and arabinoxylooligosaccharides, may be extracted from agro-residues such as sugarcane straw, wheat bran and corn stover, to serve as feedstock for postbiotic production (Hu et al. 2016; Mathew et al. 2018; Wegh et al. 2019). These biomass are usually abundant and cost-effective substrates to produce postbiotics at a larger scale (Knob et al. 2014). Following fermentation, the choice of the subsequent processing steps largely depends on the metabolites of interest, which could be the intracellular or extracellular metabolites or a combination of both. The major difference between the processing of intracellular and extracellular postbiotics lies in the initial cell lysis required for harvesting intracellular metabolites; this lysis step is s not applicable in the latter since all the metabolites are secreted into the fermentation broth.

**Table 2:** Techniques involved in postbiotic processing

Category	Method	Agents/techniques used	Principle	Reference
Extraction methods	Chemical – mediated	Sodium dodecyl sulphate (SDS), hydrochloric acid, polar solvents (methanol, ethanol, and water), non-polar solvents (hexane and chloroform)	Specific chemicals are used to disrupt cell walls, thereby releasing bioactive compounds	Mishra et al. 2024a
	Enzyme – mediated	Lipases, proteases, glycosidases	Specific enzymes are used to disrupt cell walls, thereby releasing bioactive compounds	Mishra et al. 2024a
	Mechanical	Bead beating and ultrasonication	Physical force breaks down cell structure to release bioactive compounds	Avramia and A Mariei 2022
Downstream processing	Centrifugation	Differential centrifugation	Separates bacterial cells from cell-free supernatants containing postbiotic metabolites	Nealon et al. 2024
	Chromatography	TLC, HPLC, GC	Separation of postbiotics based on the interaction between the stationary and mobile phase	Mishra et al. 2024a; Nealon et al. 2024
	Lypholization	Freeze dryer	Concentration using non-thermal approach	Nataraj et al. 2020
	Filtration	Microfiltration and nanofiltration	Filters are used to remove larger particles whilst allowing smaller molecules to pass through	Nataraj et al. 2020

The various techniques for cell lysis or disruption include enzymatic, chemical, and mechanical means, each with its unique advantages and disadvantages (Grigorov et al. 2021). For instance, mechanical methods such as pounding, sonication, and homogenisation are rapid, and quite adaptable to a variety of cells, however, they often require heat-generating equipment which could destabilise heat-labile metabolites (Sun et al. 2023). In comparison, enzymatic methods are slower and more expensive, but they are more likely to preserve the integrity of sensitive metabolites under moderate operating conditions (Abbasi et al. 2021). On the other hand, chemical methods, which are effective and readily scalable for commercial purposes, have been demonstrated to often cause denaturation of postbiotic protein components and are also associated with various safety risks (Gomes et al. 2020).

Centrifugation is the common starting point in the processing of all types of postbiotics, and it has been documented for its user-friendliness, enhanced extraction efficiency, and high recovery rates (Thorakkattu et al. 2022). Through centrifugation, cells/cell debris are separated from the metabolites which are subjected to further processing (Paul Beulah And Rajasekar, 2023). Subsequently, filtration may be employed to ensure the total separation of microbial cells from the supernatant-containing metabolites, thus avoiding interference with downstream processing and facilitating high metabolite recovery (Lee et al. 2019b).

Păcularu-Burada and Bahrim (2021) highlighted the utilisation of precipitation to concentrate accessible proteins inside postbiotic preparations, as well as to improve purity. Dialysis has also been noted to be a gentle method that allows for the selective removal of undesirable molecules and can be used to maintain the functionality and integrity of sensitive metabolites (Rad et al. 2022). Additional processing of postbiotic metabolites may also include evaporation and/or lyophilisation, which serve concentration purposes, facilitate stability during storage and prepare the functional biotics for downstream processes such as encapsulation, granulation, compaction, and spray drying (Fang And Bhandari 2017). However, during evaporation and lyophilisation, the removal of the liquid constituents may lead to the loss of volatile compounds which are usually crucial for the functionality and bioactivities of the postbiotics (Hijová, 2024; Mishra, et al. 2024a).

**Fig. 1:**
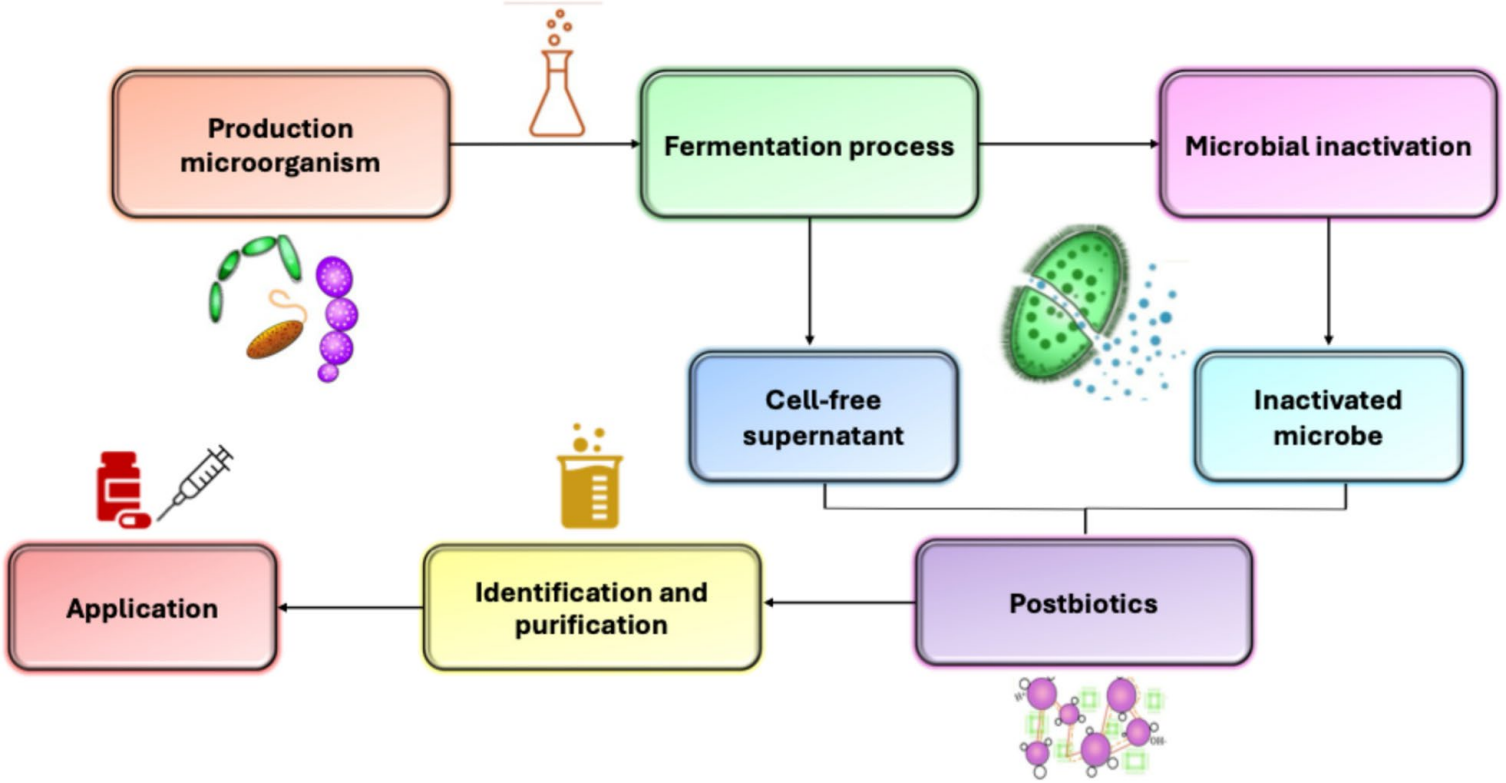
Process flow of postbiotic production

The comprehensive evaluation and effective utilisation of postbiotic preparations cannot be said to be complete without the analysis of the preparations using appropriate analytical methods. The identification of postbiotic metabolites is typically achieved via chromatographic methods such as high-performance liquid chromatography (HPLC) and gas chromatography (GC), however, it is believed that basic techniques such as thin layer chromatography (TLC) or column chromatography may also provide some preliminary insights when required (Zhou et al. 2024). Furthermore, analytical techniques such as nuclear magnetic resonance (NMR) spectroscopy (Balaguer et al. 2022), mass spectrometry (MS) (Zhou et al. 2024) and Raman spectroscopy (Aguilar-Toalá et al. 2020) have also been instrumental in characterising the biochemical composition of postbiotics. It was also noted that combining complementary analytical techniques such as spectroscopic analysis and chromatographic separations, often yields a more comprehensive description of the postbiotic profile (Segers et al. 2019). However, further downstream processing and/or modification of postbiotics is largely dependent on their final and specific industrial application. For instance, postbiotics have been used to supplement fermented foods such as kombucha, sauerkraut and yoghurt in a process known as fermentation product integration (Prajapati et al. 2023). In another instance, the synergistic effect of Fig. 1.

### Biochemical composition of postbiotics

The chemical profiles of postbiotics are essential in supporting the biological activities initiated by these compounds (Moradi et al. 2019). Furthermore, it is imperative to understand the biochemical nature of postbiotics, which plays significant roles in their absorption, metabolism, excretion and distribution across the hosts’ organs and tissues. The biochemical composition of postbiotics from different microbial sources varies as much as the metabolic capabilities of the microbial sources and is also dependant on the nature of the growth/fermentation medium (Aguilar-Toalá et al. 2018). Although postbiotics contain a wide range of metabolites in different proportions, studies have shown that the most active of the metabolites can be mainly categorised into short-chain fatty acids (SCFAs), enzymes, organic acids, lipids, polysaccharides, peptides, teichoic acids and peptidoglycan. Furthermore, vitamins (including vitamin B) and folic acid have also been found as key components in quantifiable amounts in various postbiotic preparations (Zakrzewska et al. 2022). The wide range and diversity of compounds found in various postbiotic preparations have been highlighted in the previous section of this review (Table 1); it must, however, be noted that these compounds, when consumed in their purified individual forms, can no longer be considered as postbiotics.

### Short-chain fatty acids

Short-chain fatty acids (SCFAs) are saturated aliphatic organic acids that consist of one to six carbons, with acetate, propionate and butyrate being the most common in the human body (Markowiak-Kopeć and Śliżewska, 2020). They are considered the most abundant postbiotic components produced during microbial fermentation, and they have been demonstrated to be an important regulator of human GIT homeostasis (Ragavan And Hemalatha 2024). In addition, they have been reported to possess many physiological functions such as antimicrobial, immunomodulation and antioxidant effects (Nataraj et al. 2020). For example, SCFAs such as butyrate have been proposed to prevent the growth of pathogenic microbes, and their presence in stools has been used as an indicator of gut health status (Anand et al. 2016). Furthermore, the immunomodulatory activity of a commercialised postbiotic, Hylak® forte, which contained a medley of metabolites from four bacterial strains, *Lactobacillus acidophilus* DSM 4149, *Lactobacillus helveticus* DSM 4183, *Escherichia coli* DSM 4087 and *Enterococcus faecalis* DSM 4086, was mainly attributed by its SCFAs (Patil et al. 2019). In another study, Yilmaz et al. (2022) investigated the antimicrobial activity of postbiotics derived from four LABs, namely, *Leuconostoc mesenteroides* subsp. *cremoris*, *Pediococcus acidilactici*, *Lactococcus lactis* subsp. *lactis* and *Streptococcus thermophilus*, and identified acetic acid, propionic acid and butyric acid as prominent organic acids. It has been highlighted that these acids are effective acidifying agents, capable of lowering the pH and thereby reducing the survivability of non-acid-tolerant pathogens via pH-mediated stress and disruption of microbial homeostasis (Kareem et al. 2014). According to Mishra et al. (2024b), SCFAs may also attenuate inflammation by decreasing the generation of reactive oxygen species (ROS) as well as proinflammatory cytokines.

### Polysaccharides and peptidoglycans

Polysaccharides and peptidoglycans are key components of bacterial cell walls and are found in significant quantities in postbiotic preparations as exopolysaccharides (Abbasi et al. 2023). The polysaccharides are either homopolysaccharides or heteropolysaccharides, and their specific properties vary depending on the producing organism as well as the production conditions (Bhatia et al. 2024). For example, dextran, a homopolysaccharide made of α-(1, 6)-linked d-glucopyranose- was found to be a major component of *Leuconostoc mesenteroides* postbiotics and it demonstrated significant anti-inflammatory properties. Similarly, homopolysaccharides produced by *Weissella*, *Leuconostoc*, *Lactobacillus*, and *Pediococcus* species have been noted to elicit immunomodulatory and antimicrobial effects (Guérin et al. 2020). Polysaccharides detected in postbiotics have been demonstrated to include oligosaccharides such as 3′-galactosyl lactose (Toca et al. 2023), panose, and maltotriose (Zarour et al. 2024). In addition to polysaccharides, various peptidoglycans have also been identified as components of postbiotics. Peptidoglycans, which are typically composed of alternating N-acetylglucosamine (GlcNAc) and N-acetylmuramic acid (MurNAc) residues, are integral to cell wall integrity, shape, and rigidity, especially in *Lactobacillus* strains and in other gram-positive probiotics (Zheng et al*.* 2024). These compounds have also been described for their bioactivities; for example, peptidoglycan from *Lacticaseibacillus rhamnosus* GG was shown to enhance immune responses and provide anti-inflammatory benefits (Salva et al. 2021). Similarly, *L. plantarum* and *Bifidobacterium bifidum* produced peptidoglycans with similar health-promoting effects (Semenov, 2021). It is believed that other co-polymers in peptidoglycans contribute largely to their health benefits. According to Kolling et al. (2018), their immunogenic properties may be attributed to the fact that they are cross-linked by short peptide chains. For instance, *L. rhamnosus* peptidoglycan, made up of alternating GlcNAc and MurNAc units crosslinked by peptide chains, enhanced immune function while also inhibiting pathogenic bacteria (Walter & Mayer, 2019). The beneficial effects of monosaccharides that are found in postbiotics, such as arabinose, fructose, galactose, galacturonic acid, glucose, mannose, rhamnose, and xylose have also been highlighted. For example, galacturonic-rich polysaccharides (GAPs) produced by species of *Lactobacillus* and *Leuconostoc* were described to modulate the gut microbiome and immune system (Zeidan et al*.* 2017). Furthermore, these bacterial-derived exopolysaccharides can also showcase significant antibacterial, antioxidant and antidiabetic activity. In an investigation conducted by Aliouche et al. (2024), exopolysaccharides derived from *Lactiplantibacillus plantarum* O7S1 showed significant antidiabetic activity denoted by the percentage inhibition of α-amylase at 71.57%. The postbiotic exopolysaccharides can also be utilised to reduce intestinal inflammation.

### Enzymes and peptides

It is believed that the biological activities of postbiotic preparations may be partly due to their enzyme components. Recently, the ability of *L. plantarum* to scavenge free radicals, both in vivo and in vitro, was ascribed to the presence of antioxidant enzymes such as superoxide dismutase (SOD), catalase (CAT) and glutathione/glutathione reductase (Abdel Tawab et al. 2023). Similarly, postbiotic enzymes, such as glutathione peroxidase (GSH-px), SOD, nicotinamide adenine dinucleotide (NADH)-oxidase and NADH-peroxidase, were also reported to exhibit remarkable antioxidant and antimicrobial effects (Aguilar-Toalá et al. 2018). For example, GSH-px and CAT enzyme activities were assessed by Alan et al. (2022), who highlighted the beneficial antioxidant system functions of the probiotic microorganism *Leuconostoc pseudomesenteroides* from which the postbiotic was derived. Cell-free extracts (postbiotic) of *Lacticaseibacillus paracasei* were also shown to exhibit considerable lipolytic activity at 2395 μg/g and biochemical profiling revealed the presence of antioxidant enzymes SOD, CAT and GSH-px (Osman et al. 2021). Furthermore, many postbiotic formulations contain β-galactosidases, also known as lactase, which significantly ameliorates lactose intolerance in affected individuals (Jansson-Knodell et al. 2022). Peptides, which are important components of postbiotics, are chains of amino acids produced by microorganisms; they vary widely in their sizes, specific sequences, and biological activities (Chaudhary et al. 2024). They vary from dipeptides to long polypeptide chains. For example, *L. rhamnosus* GG secreted the peptides NPSRQERR and PDENK, which have been recognised as postbiotic peptides (Kathayat et al. 2021). The majority of these peptides possess either gut-health-promoting and/or antimicrobial activities. For instance, *Limosilactobacillus reuteri* postbiotics were observed to contain various bioactive peptides that support gut microbiota balance (Peluzio et al. 2021). Furthermore, Roux et al. (2022) found that postbiotics derived from *S. thermophilus* contained peptides that can facilitate lactose digestion and promote gut health while also supporting immune system functions. Specific examples of these peptides which have been made popular for their antimicrobial activities against different pathogens include nisin which is a 34 amino acid peptide from *Lactococcus lactis*; lactacin B, a small peptide from *L. acidophilus*; and enterocin, a 3–5 kDa peptide from *Enterococcus faecium* (Vukomanović et al. 2017; Abd-Elwahed et al. 2023). In addition, bacteriocin 29, with a molecular weight of 6 kDa, from *L. plantarum* also demonstrated antimicrobial activity (Moradi et al. 2020).

### Teichoic acids

Teichoic acids are anionic polymers found in the cell walls, capsules, and membranes of most Gram-positive bacteria and are typically composed of glycerol phosphate or ribitol phosphate (Balaguer et al. 2022). These compounds, which are considered polysaccharide derivatives, exhibit promising immunomodulatory properties (van der Es et al. 2017). As key components of postbiotics, these acids are metabolites or cellular elements derived from probiotics; thus, many probiotic bacteria, including species of *Lactobacillus* and *Bifidobacterium*, produce teichoic acids with health benefits. Teichoic acids are classified into two major types, lipoteichoic acid and wall teichoic acid, both of which have significant bioactivities in immune regulation and gut colonisation, especially in *Lactobacillus* species (Shiraishi et al. 2016). For instance, teichoic acids in a postbiotic from *L. rhamnosus* GG modulated immune responses and reduced inflammation in a mouse model of colitis. Similarly, lipoteichoic acids from *Lactobacillus plantarum* was reported to elicit anti-inflammatory activities on human intestinal epithelial cells by counteracting interleukin-8 production (Noh et al. 2015). Furthermore, lipoteichoic acid from *Lactiplantibacillus plantarum* MTCC 5690, *Limosilactobacillus fermentum* MTCC 5689 and *L. rhamnosus* GG have been noted to perform various beneficial roles in host immune systems (Pradhan et al. 2023).

### Biological activities of postbiotics

**Fig. 2:**
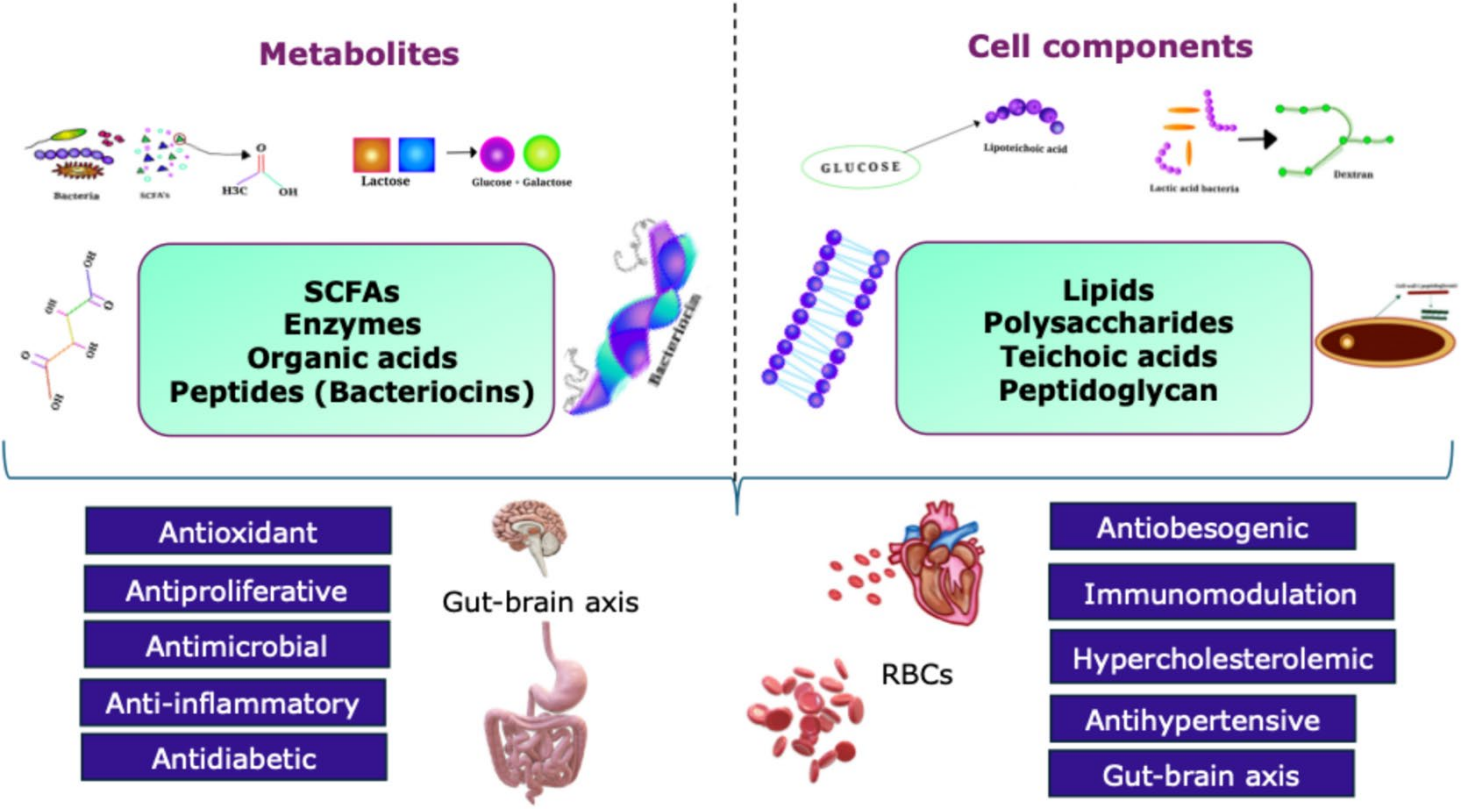
The beneficial bioactivities of postbiotics

Postbiotics not only influence the human GIT and promote gut health but can also offer potential benefits that can alleviate and prevent diseases linked to the heart, liver, and pancreas, to name a few (Rad et al. 2021b; Rad et al. 2022). A large number of in vitro and in vivo studies have demonstrated the ameliorative effects of postbiotics against various health conditions, including infections, cardiovascular diseases, diabetes, hypertension, and skin health (Fig. 2). These health benefits are mediated by various mechanisms such as alteration of the gut microbiota, enhancement of immune responses, antiproliferation, antioxidation and the direct induction of apoptosis (Chuah et al. 2019). The modulatory effects of postbiotics are due to their metabolic byproducts as well as their cell components as highlighted earlier (Tables 1) in this article.

### Antioxidant

Various postbiotic preparations have been reported, both in vitro and in vivo, to exhibit strong antioxidant properties (Qiao et al. 2024; Yousefvand et al. 2024). It is believed that these antioxidant capabilities may facilitate the recovery and balance of gut microbiota from dysbiosis, typically caused by the various ingested substances such as colourants, preservatives and medicinal substances which exhibit some antimicrobial effects (Beres et al. 2020). In addition to these extraneous stressors, the human microbiota is also exposed to endogenous free radicals which further impair health, causing oxidative stress leading to a number of chronic diseases such as cardiovascular, diabetes and neurodegenerative diseases (Blazheva et al. 2022).

The antioxidant capabilities of postbiotics are largely due to the cellular antioxidant enzymes glutathione peroxidase (GPx) and catalase (CAT), which are the first line of cellular defence (Alan et al. 2022). A study by Kang et al. (2021) highlighted the antioxidant potential of heat-inactivated postbiotics derived from *Bifidobacterium bifidum* MG731 possessing high 2,2-diphenyl-1-picrylhydrazyl (DPPH) free radical scavenging activity (90%). Similarly, *B. lactis* MG741- derived postbiotics showed remarkable ABTS radical scavenging activity (99.5%), which is attributed to the postbiotic antioxidant enzymes. Furthermore, the use of postbiotics in cosmetics has become popular due to their radical scavenging activities against skin-damaging reactive oxygen species (ROS). This was highlighted by Lee et al. (2024), where the antioxidant and wrinkle-improving activities of *Levilactobacillus brevis* BK3-derived postbiotics were assessed and showed a high antioxidant capacity of 30.97% and 46.65% for both DPPH and ABTS radical scavenging activities. These antioxidant postbiotic capabilities extend far beyond the skin-surface level and have also been reported to enhance ruminal barrier function. This was observed by Izuddin et al. (2020), where postbiotics produced from *L. plantarum* had shown improved antioxidant activity with increased glutathione peroxidase (GPX) present in the post-weaning lamb ruminal serum, thus highlighting its potential in maintaining ruminal barrier function. Additionally, antioxidant postbiotics have also been reported to mitigate the effects of ulcerative colitis. Rezaie et al. (2025) conducted an in vivo study against mice with dextran-induced colitis and showed that the mice that received the antioxidant postbiotic cocktail had a lower disease activity index with increased intestinal mass and length (p < 0.05). This highlights the potential of postbiotic formulations as a promising biotherapeutic agent with enhanced antioxidant activity across multiple target sites.

### Anti-inflammatory

Inflammation is the response of the immune system to a harmful stimulus such as injury or infection (Rivera-Jiménez et al. 2022). However, as demonstrated in many studies, such conditions can be mitigated by the administration of postbiotics. For example, Rezaie et al. (2024) investigated the effectiveness of postbiotic mixtures from various *Lactobacillus* and *Bifidobacterium* species and showed that the preparations elicited a significant reduction of pro-inflammatory cytokines and an increase in anti-inflammatory cytokines. Similarly, the consumption of *Lactobacillus delbrueckii* CIDCA 133 was observed to induce immunomodulatory actions in colitic mice by increasing the levels of anti-inflammatory cytokines TGFβ and decreasing the expression of inflammatory markers (dos Santos et al. 2024). It has been reported that the metabolites present within postbiotic formulations inhibit the growth of harmful microorganisms and reduce the elevated levels of inflammatory cytokines induced by these microbes. Chung et al. (2022) showed that postbiotic complex treatment (*Lactobacillus helveticus* HY7801 and *Lactococcus lactis* HY449) had regulated the inflammatory cytokines IL-8 while simultaneously exhibiting activity against skin pathogens *S. aureus* and *Cutibacterium acnes*. The abovementioned examples showcase the promising anti-inflammatory properties emerging from postbiotic sources, from intestinal inflammation (colitis) to skin inflammation, highlighting its potential as both a topical agent and biotherapeutic agent for oral administration whilst maintaining its overall activity.

### Antidiabetic

Postbiotics are considered one of the management strategies for diabetic complications due to the interesting interaction between human gut microbiota and the gut-brain axis, which includes the intestinal nervous system (Kim et al. 2024b). Postbiotic components, especially SCFAs such as acetate, propionate and butyrate, have been noted to alleviate type 2 diabetes by maintaining intestinal homeostasis and regulating glucose metabolism upon administration (Kim et al. 2024b). Similarly, gamma-aminobutyric acid (GABA)-rich postbiotics improved metabolic indices in diabetic mice models (Abdelazez et al. 2022). Similarly, Beteri et al. (2024) have shown that postbiotics rich in exopolysaccharides and SCFAs from *B. breve* 091109 facilitated a significant increase in GLP-1 and PYY production and consequently an increase in insulin secretion and HbA1c levels, highlighting their remarkable potential in alleviating symptoms amongst prediabetic individuals (Beteri et al. 2024). Recently, Beteri et al. (2024) utilised both galactopolysaccharides and exopolysaccharides from *Bifidobacterium breve* postbiotics in a clinical trial with prediabetic volunteers and reported a significant increase in butyrate-producing bacteria indicating its potential in managing prediabetes with minimal invasive intervention.

### Antiobesogenic activity

The ingestion of postbiotics, especially cell-free supernatants from probiotics such as *Bacillus velezensis,* has been found to elicit anti-obesogenic effects via a reduction in blood cholesterol, adipose tissue and body weight (Shin et al. 2024). In addition, postbiotics derived from the fermentation of LAB (strain LKDH5) were shown to mitigate high-fat diet-induced weight gain, adipose tissue accumulation, and elevated plasma triglyceride levels, which were all linked to favourable changes in the gut microbiota, further supporting the hypothesis that postbiotics influence metabolic health by modulating microbial communities (Youn et al. 2022). The postbiotic preparation from *Bifidobacterium infantis* was described to contain a range of short-, medium, and long-chain fatty acids, which may induce thermogenesis via the promotion of “browning” in white adipocytes (Rahman et al. 2023). Some evidence has also suggested that postbiotic supplementation may support mood, reduce fatigue, and increase athletes'readiness during exercise training (Kerksick et al. 2024). Furthermore, supplementation of postbiotics, particularly from *Lactiplantibacillus plantarum* I-UL4, in animal species such as piglets, mice, and rats has been shown to alter metabolic activity, especially glucose homeostasis (Lin et al. 2020). Studies investigating the effects of postbiotics in rats have also elucidated their role in modulating cholesterol levels, promoting growth, and influencing the pH of the gastrointestinal tract, with short-chain fatty acids, such as butyrate and propionate, being associated with improved hunger control and enhanced energy metabolism (Li et al*.* 2018; Song et al. 2019).

### Immunomodulation

The immunomodulatory ability of postbiotics has been described in various studies as having the ability to stimulate both the innate and adaptive immune systems via different biological mechanisms. (Rocchetti et al. 2024; Salva et al. 2021; Xin et al. 2024). In particular, postbiotics interact with immune cells, which, in turn, hasten the production of useful cytokines (Azad et al*.* 2018). Their immunomodulatory effects have also been shown to occur via the regulation of the Th17/Treg balance (Xin et al. 2024), enhancing the production of IL-10 and TNF-α factors, altering cytokine patterns in human macrophages, and attenuating the transcriptional activation and secretion of both TNF-α and IL-8 (Rocchetti et al. 2024). It was noted that postbiotics from *Lacticasebacillus rhamnosus*, *L. reuteri*, and *Bifidobacterium animalis* subsp. *lactis* demonstrated remarkable immunomodulatory effects and competed favourably with activities obtained from live probiotic strains (Yeşilyurt et al. 2021; Zago et al. 2021). A study by Salva et al. (2021) also highlighted that in addition to the safety of postbiotics derived from *Lacticaseibacillus rhamnosus* compared to living bacteria, the postbiotic also showed higher immunomodulatory activities. It was also suggested that increased levels of short-chain fatty acids found within postbiotics may correlate negatively with opportunistic *E. coli* and *Bacteroidetes*, thus, their gut microbiome modulation role (Szydłowska and Sionek, 2022). The postbiotics from *Lactobacillus jensenii* TL2937 were also described to reduce dextran sodium sulphate-induced colitis in mice by altering the genomic response of intestinal epithelial tissues (Sato et al. 2020).

### Antihypertensive and hypercholesterolaemic activities

Postbiotics are emerging as promising antihypertensive therapeutics as evidence has shown that postbiotics are largely responsible for the antihypertensive activity of some probiotics. The ability of *L. plantarum* to reduce blood pressure in nutritional therapy was partly attributed to its postbiotics (Robles-Vera et al. 2017). The SCFA components of postbiotics, such as butyrate and propionate, have been linked severally to blood pressure management, and it is believed that they increase endothelial function and reduce inflammation (Tain et al. 2023). According to Wang et al. (2017), sodium butyrate, an SCFA, lowers blood pressure through the renin-angiotensin system pathway, especially by inhibiting the intrarenal renin-angiotensin system which raises blood pressure in angiotensin II-induced hypertension. In the same vein, exopolysaccharides-rich postbiotics from *L. planturum* have also been highlighted for their significant antihypertensive activity (Gezginç et al. 2022). Postbiotics have also emerged as promising agents in the management of hypercholesterolemia. Studies indicate that they can effectively cut down total cholesterol and low-density lipoprotein cholesterol levels in a similar fashion to probiotics. In particular, postbiotics from *Lactobacillus acidophilus* CL1285, *L. plantarum*, and *L. reuteri* have demonstrated significant cholesterol-lowering capabilities (Frappier et al. 2022). The mechanisms behind these cholesterol-lowering effects are multifaceted. For instance, muramyl dipeptide (MDP), a bacterial cell wall component, plays a role in lipid metabolism regulation and stimulates immune defences (Park et al. 2017). Also, postbiotic metabolites like SCFAs inhibit cholesterol synthesis and enhance bile acid excretion, while bacteriocins also lower their cholesterol levels (Wegh et al. 2019).

### Antimicrobial and antiproliferative activity

Postbiotics possess a mixture of bioactive metabolites responsible for bacterial lysis, including bacteriocins, organic acids, hydrogen peroxide and fatty acids, all with prominent antimicrobial potential (Moradi et al. 2019). The composition of these soluble bioactive antimicrobial agents and their effectiveness varies depending on factors such as the strain utilised and fermentation conditions (Ooi et al. 2021). For example, Tong et al. (2023) assessed the *E. coli* inhibitory activity of postbiotics obtained from solid state fermentation, which varied depending on the combination of strains, incubation temperature, and water content of the fermentations. The antimicrobial action of postbiotics against fungi has also been established. For instance, Ebrahimi et al. (2021) reported the anti-mycotoxigenic capabilities of *Apilactobacillus kunkeei* postbiotics as well as in situ inhibitory effect against *Candida albicans*. Given the latest trends in functional biotics, postbiotics may be a promising alternative to counter antibiotic resistance in the food and pharmaceutical industries (Hossain et al. 2021). Uncontrolled cell proliferation has been identified as one of the hallmarks of the various types of cancers in the human body. A large number of probiotics such as LAB have been reported previously to impact the carcinogenesis process associated with various cancers (Motevaseli et al. 2017). However, postbiotics derived from various probiotics have efficiently shown their therapeutic potential in new cancer treatment strategies due to their significant cytotoxicity (Dameshghian et al. 2024). The mechanism of antiproliferative action of postbiotics from several LAB was determined by Nowak et al. (2022), whereby stronger inhibitory activity was found against Caco-2 and HeLa cells; cellular apoptosis was also recorded in the supernatants of both *L. plantarum* and *Levilactobacillus brevis*. Similarly, the impact of postbiotics derived from *B. breve* was notably effective against HT-29 cells, highlighting its anti-proliferative, anti-migration and apoptosis-related effects (Erfanian et al. 2024).

### Industrial applications of postbiotics

Postbiotics, as the metabolic byproducts or components of probiotic microorganisms, offer a novel avenue for developing innovative products with enhanced functionalities and health-promoting properties. As earlier stated, various investigations have demonstrated the effectiveness and efficacy of postbiotic preparations at levels that are commensurate with their more popular counterparts, viz., probiotics and prebiotics. In this regard, the applications of postbiotics-based products in various industries including food and beverage, pharmaceuticals, veterinary, and cosmetics, continue to garner considerable interest due to their potential impact on various industrial sectors. Thus, the current and potential industrial applications of postbiotics, as well as their role in fostering the development of value-added products and addressing the evolving consumer demands for natural, functional, and sustainable solutions, are reviewed in this section.

### Food and beverage

The use of postbiotics as natural or bio-preservatives, food supplements, postbiotic-enriched functional foods, and bioactive compounds in food formulations aligns with the growing consumer preference for clean-label products and the industry's pursuit of sustainable and eco-friendly ingredients (Poeta et al. 2023; Mishra et al. 2024a). This is in light of increasing concerns about the potential long-term effects and individual sensitivities associated with conventional food preservatives such as butylated hydroxyanisole (BHA) and butylated hydroxytoluene (BHT), as well as the nitrites, nitrates and sulphites (Lalani et al. 2024). For instance, cell-free supernatant from a *L. plantarum* strain was demonstrated to serve as an effective biopreservative for chicken breast fillets which had been contaminated with *Enterococcus faecium* 711, maintaining the integrity of the food product for over a week (da Silva Sabo et al. 2017). Similarly, postbiotic preparation derived from *Lacticaseibacillus rhamnosus* EMCC 1105 at a dose of 100 mg/g significantly reduced *Clostridium perfringens* in minced chicken (Hamad et al. 2020). Postbiotics have also shown potential in preserving non-meat products, as demonstrated by the preservative effect of cell-free supernatant from *Lactiplantibacillus plantarum* YML007 on soybean grains (Ahmad et al. 2013). In addition, postbiotics have been used to supplement fermented food products, such as yoghurt, to enhance the organoleptic properties and nutritional value of the final product (Huang et al. 2020). This is in accordance with the hypothesis that the health promoting effects of fermented foods, including kombucha, kimchi, kefir, tempeh and yoghurt, might be due to their postbiotic conten3t (Gill And Staudacher 2023; Isaac-Bamgboye et al. 2024). In one notable example, incorporating *Lactarius volemus* Fr. postbiotic extracts into probiotic yoghurt significantly improved water-retention capacity, essential amino acid content and the overall quality of the product (Huang et al. 2020). Postbiotics have also been demonstrated to enhance the functional properties of food packaging materials, preventing food spoilage and extending shelf life in the process (Shafipour Yordshahi et al. 2020). These enhanced functional properties are believed to be mainly due to the antimicrobial peptides, bacteriocins and organic acid components of the various postbiotics (Moradi et al. 2020). In another study, impregnating a biobased packaging material made from bacterial nanocellulose with lyophilised postbiotics of *L. plantarum* at a concentration of 21.21% decreased the total mesophilic and psychrophilic count as well as lipid peroxidation of the enclosed meat products (Shafipour Yordshahi et al. 2020). The supplementation of chitosan with *Pediococcus acidilactici* postbiotic has also reduced the total viable count, LAB, and psychotropic bacteria in the enclosed chicken breast fillets, extending the shelf-life by approximately 12 days (İncili et al. 2021).

### Pharmaceutical industry

The pharmaceutical sector has witnessed a surge in interest in leveraging postbiotics for the development of novel therapeutic agents and drug delivery systems (Heniedy et al. 2024), as postbiotics exhibit a diverse range of bioactivities, as earlier reviewed in this article. In particular, the antiproliferative activity of postbiotics from various sources has raised considerable interest in their potential for cancer management (Rad et al. 2021a). According to Aghebati-Maleki et al. (2021), postbiotics may act via the modulation of immune responses, induction of intrinsic and extrinsic apoptotic pathways, inhibition of cancer invasion, reduction of angiogenesis, decrease in cell proliferation, as well as delay of cell cycle progression. For example, postbiotics derived from *Lactobacillus delbrueckii* OLL1073R-1 enhanced the proliferation of T and NK cells and regulated the immune response by interacting with dendritic cells and macrophages, demonstrating their ability to resist tumour cells (Makino et al. 2016). Additionally, postbiotics derived from different strains of *L. plantarum* exhibited significant antiproliferation against human colorectal adenocarcinoma cells (HT-29) and some suspension cancer cells (K562 and HL60) (Chuah et al. 2019). Postbiotics also exhibit notable antimicrobial activities, thus raising possibilities of their utilisation in the formulation of antibiotics in the pharmaceutical industry (Hossain et al. 2021, Ishikawa et al. 2021; Azami et al. 2022). Their antimicrobial effect has been majorly attributed to the ability of different components to impede quorum-sensing activities, inhibiting social contact and communication between pathogens, and thereby interrupting biofilm production and dispersing preexisting biofilms (Ishikawa et al. 2021; Azami et al. 2022). Interestingly, postbiotics derived from *L. plantarum* and *Latilactobacillus curvatus* inhibited the growth and biofilm-forming capabilities of the highly pervasive food-borne bacteria, *Listeria monocytogenes* (Hossain et al. 2021). There are recent instances where postbiotics such as those of *Lacticaseibacillus rhamnosus* GG, were incorporated into supplements to provide protection against oxidative stress and diarrhoea in human enterocytes induced by SARS-CoV-2 derived antigens (Poeta et al. 2023). Increased focus has also been placed on designing effective postbiotic delivery systems for increased efficacy and stability, e.g., nanoformulations and nanocarriers ). In this regard, nanoparticles or liposomes have been recorded to protect postbiotics from degradation and facilitate controlled release at the intended site (Liang and Xing 2023; Zhang et al. 2023). For example, *L. paracasei* postbiotic encapsulated by nanoparticles demonstrated outstanding anti-gastric cancer activity (Huang et al. 2020). Furthermore, technologies such as microencapsulation and spray drying have also facilitated the integration of postbiotics into pharmaceutical formulations by enhancing their stability and controlled release profiles, as well as improving their bioavailability and therapeutic efficacy (Khani et al. 2024).

### Veterinary medicine and nutrition

Postbiotics have been noted to exhibit many beneficial effects on animal health by promoting innate and adaptative immunity and decreasing intestinal colonisation and multiplication of pathogenic microorganisms (Karuvelan et al. 2025). These make them eco-friendly components that balance animal body performance and promote the production of safe animal-based products, such as eggs and meat for human consumption (Feye et al. 2019). In animal husbandry, postbiotics have also demonstrated potential as antibiotic alternatives, growth promoters, and immunostimulants (Ibraheim et al. 2023). For example, in dairy farms, *S. cerevisiae* fermentation products (SCFP) demonstrated notable benefits for calves, including improved performance, immune system function, post-weaning body gain, and management of bovine diseases (Sivinski et al. 2022). The immunomodulatory properties of the SCFP were observed to effectively reduce the occurrence of bovine respiratory disease in newborn calves, subsequently decreasing mortality rates in their population (Sivinski et al. 2022). Using postbiotics instead of commercial antibiotics to treat clinical and subclinical bovine mastitis was recently identified as a safe approach against antibiotic resistance (Mathur et al. 2022). Postbiotics derived from *Latilactobacillus sakei* EIR/CM-1 strain found in cow milk demonstrated potent antibacterial and antibiofilm activities against cow mastitis pathogens, including methicillin-resistant *Staphylococcus aureus* ATCC 43300 and *Streptococcus agalactiae* ATCC 27956, achieving more than 70% reduction in bacterial load. This holds promising prospects for cow farmers and veterinarians in effectively controlling cattle mastitis (Sevin et al. 2021). Consequently, postbiotic-based products, such as NutriTek® LS, Dia-V™ SC, and LiquiPro™ SC, present a promising option for farmers to control the prevalence of cow mastitis and other teat and nipple infections, which are considered economically significant diseases (Vailati-Riboni et al. 2021). Incorporating postbiotics into aquafeed formulations also showed positive effects on the growth performance and disease resistance of aquatic species, thereby contributing to sustainable aquaculture practices and the production of safe and quality seafood products (Tao et al. 2024). For instance, postbiotics derived from *S. cerevisiae*, used as feed additives in aquaculture fish and shrimp farms, have been shown to support digestive tissue integrity and increase survivability and production yield (Wang et al. 2021).

### Cosmetic formulation

**Fig. 3:**
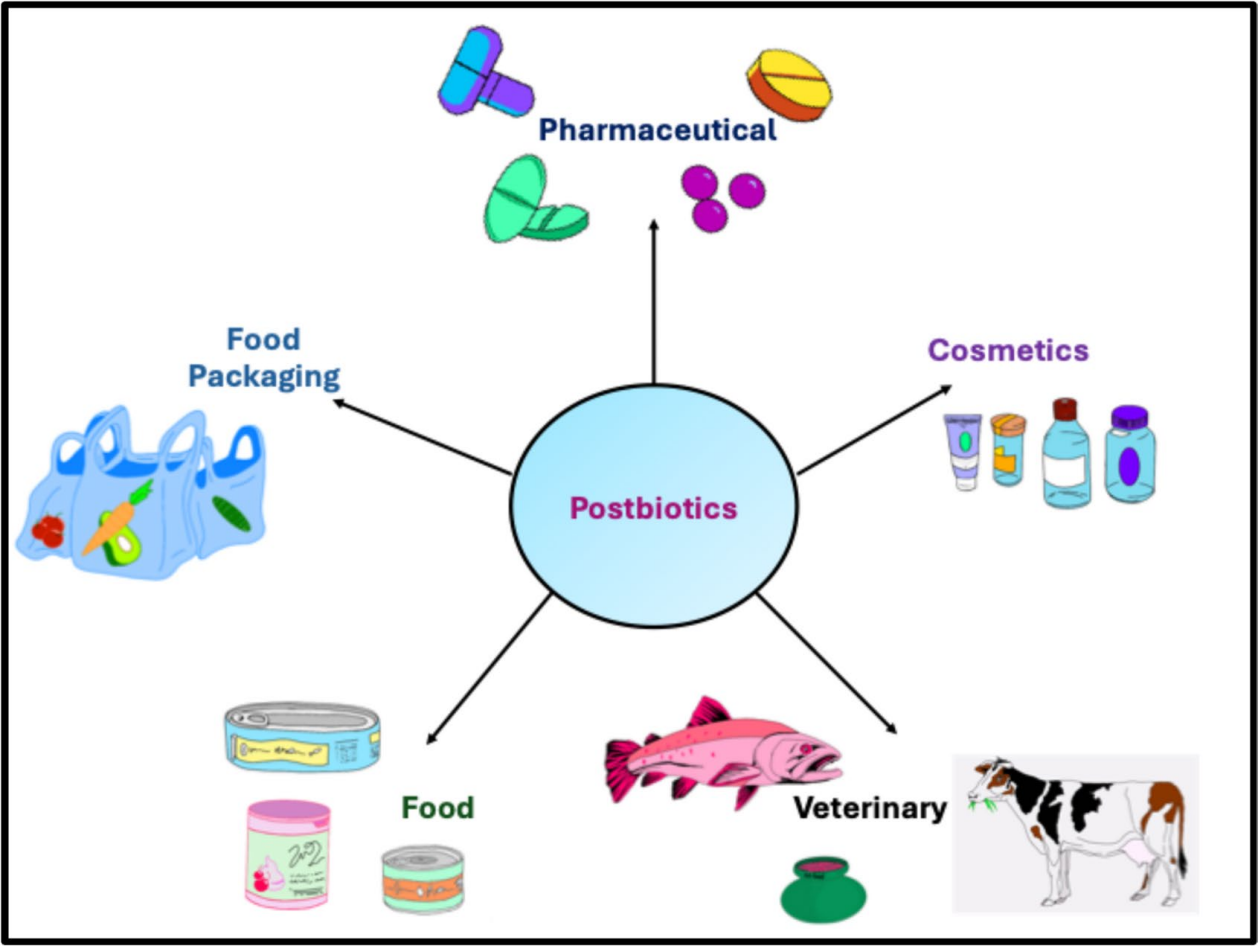
Applications of postbiotics in various industries

Recent trends have shown the applicability of postbiotics as active ingredients in cosmetics and personal care products with enhanced health benefits and microbiome-friendly properties (Majeed et al. 2020; Machado et al. 2023). It has been shown that the incorporation of postbiotics into skincare formulations, such as creams, postbiotic-infused serums, and lotions, offers the potential to modulate the skin microbiota, support skin barrier function, mitigate inflammatory skin conditions, alleviate skin sensitivities, improve microbial facial diversity and overall complexion (Kim et al. 2023). For instance, BiomeRenew™ and Lactosporin® are postbiotics-dependent skin care products (extracellular metabolites) derived from the probiotic strain *Bacillus coagulans* and used as efficient anti-acne formulations due to their ability to reduce sebaceous secretion by their 5-alpha reductase inhibition (Majeed et al. 2020). Postbiotic preparation of *Vitreoscilla filiformis* applied topically to the skin improved barrier function, promoted the growth of endogenous bacteria, and facilitated the restoration of a healthy skin microbiome (Gueniche et al. 2021). The extract from *Vitreoscilla filiformis* has been utilised as an anti-acne treatment in the La Roche-Posay (LRP) brand due to its antibacterial and antioxidant effects against acne-related bacteria and chronic inflammatory skin conditions (Heniedy et al. 2024). Additionally, the use of postbiotics in hair care products, including shampoos and hair conditioners, has been explored due to their potential to restore scalp microbiome balance and address scalp conditions, contributing to the development of holistic hair care solutions (Yoon et al. 2022). For instance, a shampoo containing heat-inactivated *Lacticaseibacillus paracasei* GMNL-653 postbiotic improved human scalp health by regulating the scalp microbiome (Tsai et al. 2023). Specifically, the heat-killed GMNL-653 was able to co-aggregate with the human scalp commensal fungus, *Malassezia furfur*, in vitro, and the GMNL-653-derived lipoteichoic acid inhibited the biofilm formation of *M. furfur* on Hs68 fibroblast cells in a dose-dependent manner. It has also been indicated that postbiotic preparations of *L. chungangensis* CAU 1447 have a beneficial effect on wound healing. In a study involving type 1 diabetic mice, wound dressings infused with these postbiotics significantly reduced wound size and enhanced the expression of healing-related cytokines, growth factors, and chemokines. Key compounds in the preparation, such as palmitic acid and palmitoleic acid, contributed to increased anti-inflammatory activity and inhibited pro-inflammatory cytokines, thereby promoting the wound-healing process (Nam et al. 2021). Similarly, using postbiotics in oral care products, such as toothpaste and mouthwash, holds promise for promoting oral microbiome balance and oral health maintenance. Notably, postbiotics from *L. paracasei* were demonstrated as key ingredients in some commercial oral products, specifically toothpaste and mouthwash, reducing halitosis, and regulating the oral microbiome while also exhibiting anti-bacterial activities against oral pathogenic bacteria (Nam et al. 2021). Fig. 3.

### Commercial status of postbiotics

Currently, the postbiotic supplements market is valued at approximately USD 11 million, and it is projected to increase a CAGR of 10.9% to ~ USD 30 million by 2034 (Future Market Insights, 2024). It is believed that this projected 200 percent increase in the market value would be propelled by raising awareness about the health benefits of postbiotic products, especially as more evidence has linked overall health with the gut microbiome, as well as the demand for naturally-derived products. Based on a market report that estimated the total functional biotics market, which comprises probiotics, prebiotics, and postbiotics, between 2024 and 2030 to be USD 57 million, it can be inferred that the postbiotic market accounts for approximately 20% of the total share (Precision Business Insights, 2023). The major players in the postbiotic-based markets have been noted to include Archer-Daniels-Midland Company (USA), AB-BIOTICS, S.A. (Spain), Adare Biome (France), Bioflag Group (Taiwan), Cargill Incorporated (USA), Danish Agro (Denmark), Lactobio A/S (Denmark), GeneFerm Biotechnology Co. (Taiwan), Kirin Holdings Company, Limited (Japan), KOREA BeRM Co. Ltd. (South Korea), Lesaffre (France), MCLS Europe (Netherlands), Mitsubishi Corporation Life Sciences Ltd. (Japan), Postbiotica S.R.L. (Italy), Probiotics Australia Pty (Australia) and Sabinsa Corporation (USA). A critical review of the regulation and guidelines of the leading regulatory authorities around the world, including the US Food and Drug Administration, European Food Safety Authority, Japanese Pharmaceutical and Food Safety Bureau as well as the UK’s Medicines and Healthcare Products Regulatory Agency, revealed that the regulatory landscape of postbiotics is relatively undefined. This observation poses significant challenges for standardisation, manufacture, growth, and innovation in postbiotic-based products. Furthermore, some insights were also gained into the commercial landscape of postbiotics by the analysis of the currently available patents. The information obtained from patent databases, including the Canada Patent Office, the European Patent Office, the French National Institute of Industrial Property, the Korea Intellectual Property Rights Information Service, the China Patent, the Taiwan Intellectual Property Office, the Russian Federal Service for Intellectual Property and the US Patent Office, also highlights the increasing focus on the utilisation of postbiotics for personalised health solutions, particularly targeting gut health through innovative delivery systems (Table 3). There are currently more than 1000 patents on postbiotic-based products, which vary from animal feed, antibiotics, food supplements, and cosmetic formulations, to mention a few. It was also observed that the major patent assignees were business establishments and not educational or research institutions, and the leading companies were identified as Seed Health Inc (USA), ATA BIO Technology Food Medicine Cosmetics Industry Trade Ltd. (Turkey), Jihaesi Life Science Co. Ltd. (South Korea), Guangzhou Zhengming Biotechnology Co. Ltd. (China), Can Technologies, Inc. (USA), Sichuan Gaofuji Biotechnology Co. Ltd. (China), and Fonterra Co-operative Group Limited (New Zealand), arranged in descending order with each company accounting for between 1% and 2.3% of the total patents.

**Table 3:** Patents related to postbiotics across different industries

Patent no	Invention	Highlight(s)	Assignee	Reference
AU2015296538B2	Animal feed compositions and feed additives	Animal feed compositions and additives containing a mixture of pre-, pro- and post-biotic materials in promoting animal growth, health, and nutrition	BiOWiSH Technologies Inc	Joella et al. 2019
CN117180318A	Metagen composite fermentation liquor, preparation method and application thereof	Metagen composite fermentation liquor prepared from *Rhizoma polygonati* polysaccharide extract and *Lactobacillus reuteri* WX-94, in the production of medicine to improve glycolipid metabolism disorder function	Shenzhen Huayouyi Biotechnology Co Ltd. Shaanxi Normal University	Shi et al. 2024
CN115927122A	Post-growth hormone prepared from *Lactibacillus paracasei* and having the effects of promoting host hyaluronic acid (HA) synthesis and enhancing HA application	A metazoan from cheese *L. paracasei* CCFM1293, promoting host HA synthesis and enhancing its application effect, belongs to the technical field of microorganisms and medicines	Jiangnan University	Cui et al. 2023
CN115463159A	Application of *Akkermansia* and *Akkermansia* postbiotic in preparation of medicine or health-care product for preventing or treating hepatic steatosis	The invention uses *Akkermansia mucinia* and *Akkermansia metaplastron* to create a medicine for liver lesions, with live and inactivated forms showing efficacy and potential for widespread use	First Affiliated Hospital of Jinan University	Justge And Yu 2022
CN117736942B	Fermented *Lactobacillus mucilaginosus* JYLF-315 for improving skin ageing, and metagen preparation and application thereof	The study on mice shows that the *L. mucilaginosus* JYLF-315 post-biotics effectively improve skin ageing by affecting skin appearance, selenium concentration, estrogen and skin ageing-related enzyme content in muscle tissue and serum	Shandong Zhongke Jiayi Bio Engineering Co Ltd	Pan et al. 2024
CN111802649B	Preparation method and application of LGG fermentation product containing immunoregulatory peptide functional component	A preparation method and application of an LGG fermentation product containing immunoregulatory peptide, morphine-like peptide functional components, using *Lactobacillus rhamnosus* LGG as a single fermentation strain	Hebei Vermont Biotechnology Co. Ltd	Ling et al. 2023
CN115414392B	Composition containing *Lactobacillus rhamnosus* JL1 metagenesis powder, preparation method and application	A method for preparing an anti-inflammatory medicament using *L. rhamnosus* JL1 postbiotic powder, and fructo-oligosaccharide compound formula	Northeast Agricultural University	Jiang et al. 2023
EP3993819A1	Quorum-sensing inhibitors and/or postbiotic metabolites and related methods	A synergistic combination comprising a quorum-sensing inhibitor, postbiotic metabolite, and antibiotic used for resensitising resistant bacteria and treating antibiotic-resistant infections like MRSA	Microsintesis Inc	Monica 2023
EP4045069A1	Fecal-derived sterile postbiotic composition and method therefore	The invention outlines methods for sterilising faecal microbiota, purifying, freezing, and drying compositions, and their use in beverages, foods, and supplements for treatment purposes	Thaena Inc	Andrea And Piper 2023
FR3133727B3	Postbiotic food additive for products obtained from cereal flour, food composition and food products comprising the same	The invention relates to a postbiotic food additive made from cereal flour, containing a blend of lysates and probiotic microorganisms	Igen Biolab Group AG	Guardia 2024
GB2620891A	*Lactobacillus plantarum* postbiotic and use thereof in products for changing intestinal microecology and reducing cholesterol	Systematic study of the therapeutic effects of heat-inactivated bacteria or lytic *L. plantarum* on high cholesterol and evaluating their impact on intestinal flora regulation and cholesterol reduction	Thankcome Biological Science and Tech Suzhou Co Ltd	Ma et al. 2024
JP2024510374A	Composition containing polysaccharide or extract derived from *Lactobacillus plantarum*	A composition containing a polysaccharide or an extract from *L. plantarum*, can suppress inflammatory reactions, adipogenesis, intracellular glucose uptake, insulin resistance, and hepatic steatosis	Neoregen Biotech	Jung And Jae 2024
JP7395622B2	Novel *Ficalibacterium prausnitzii* strain EB-FPDK11 and its uses	*F. prausnitzii* EB-FPDK11 strain culture solution, crushed product, or extract is a pharmaceutical composition used to prevent or treat inflammatory, liver, or metabolic diseases	Enterobiome Inc	Jae et al. 2023a, (Jae b)
JP7385930B2	Formulation for the treatment of inflammatory bowel diseases using whole plant fibre extract from sugarcane	The use of whole plant fibre extracts from sugarcane in individuals’ diets for the prevention and/or treatment of inflammatory bowel diseases	University of Tasmania	Eli et al. 2023
KR102332021B1	Manufacturing method for aminobiotics using medium comprising A2 beta casein, and aminobiotics prepared therethrough2332021B1	The process involves using A2 beta casein protein as a *Lactobacillus* medium, culturing it with *Lactobacillus* isolated from breast milk, and obtaining a culture product containing metabolised A2 beta casein protein	Hyundai Bioland	Minkyu et al. 2021
KR102491324B1	Method for lowering molecular weight of collagen using fermentation of *Lactobacillus rhamnosus* IDCC 3201 and complex comprising collagen and postbiotics	A method for low-molecularization of collagen using *L*. *rhamnosus* IDCC 3201 fermentation and a complex containing collagen and postbiotics	Ildong Bioscience	Suyeon et al. 2023
KR102497827B1	*Lactiplantibacillus plantarum* DCF49, a new strain derived from shindari and production method of functional postbiotics using the same	Postbiotics produced by isolating a novel *L. plantarum* DCF49 strain from shindari, a traditional food of Jeju Island food, and fermenting it under optimal conditions	Jung Co., Ltd. food	Sungsoo et al. 2023
KR102542030B1	Makgeolli-derived *Lacticaseibacillus paracasei* subsp. *paracasei* DCF0429 and functional postbiotics using the same	*L. paracasei* strains from makgeolli have high antioxidant capacity, and postbiotics made from these strains in soybean embryo medium can also exhibit excellent antioxidant properties	Jung Co., Ltd. food	Jae et al. 2023a, (Jae b)
KR102427841B1	Composition comprising complex enzymes to improve digestion and toxin excretion	An enzyme complex comprising 10 types of enzymes can be used as a main ingredient in healthy functional foods or pharmaceutical compositions,	Jaegyu Lee	Jaegyu et al. 2022
KR102578662B1	Composition for preventing hair loss and promoting hair growth comprising postbiotic components from heat-treat *Limosilactobacillus fermentum* LM1020	Heat-treated *L. fermentum* LM1020 prevents hair loss, promotes hair follicle cell proliferation, and inhibits 5α-reductase-1, growth factors FGF7, FGF10, and EGF expression	Lactomaison	Wonyoung et al. 2023
KR102513864B1	Washing machine cleaner composition comprising postbiotics component, and washing machine cleaner container taking the washing machine cleaner composition comprising postbiotics component	Postbiotics-containing washing machine cleaner includes *Lactobacillus plantarum*, *L. casei*, and *L. rhamnosus*. is environmentally friendly, with improved detergency and biodegradability	ONSAEM KOREA	Jeongju And Jung 2023
KR102463565B1	An eco-friendly laundry detergent composition containing a post-biotic component and a laundry detergent composition manufacturing device for manufacturing the same	An eco-friendly laundry detergent composition containing a postbiotic mixture formed by mixing a first, improves washing efficiency by removing contaminants	Clean Hill Inc	Lee 2022
KR102374480B1	*Lactobacillus paracasei* EPS DA-BACS promoting the growth of *Bifidobacterium* and inhibiting *Clostridium difficile* and polysaccharide therefrom	*L. paracasei* EPS DA-BACS is a postbiotic for promoting bifidobacterium growth, inhibiting harmful bacteria, and providing a prebiotic effect, promoting gastrointestinal stability and inflammation relief	Dong-Ajewak Co., Ltd	Han et al. 2022
KR102251295B1	A composition for preventing or relieving hangover of the comprising heat-killed *Lactobacillus salivarius* v133 as an active ingredient	A pharmaceutical composition to prevent hangovers and a food additive containing dead *L. salivarius* v133 strain as an active component	Medinewrol Co., Ltd., Cho Hyanghyun	Cho et al. 2021
LU502431B1	A postbiotic S515 production formula with lipid-lowering and metabolism-regulating functions and its application	A postbiotic production formula in probiotic technology regulates lipid levels and metabolism by modulating intestinal flora, inhibiting fat absorption, and reducing liver and visceral fat accumulation	Smeal Ltd	Peng And David 2023
RU2819908C1	Enzymatic method of producing lactulose-containing postbiotic product	The method involves two-stage cultivation of lactase producers, including aerobic and anaerobic stages, enzyme extraction, and lactulose biosynthesis, followed by fermentation and concentration	Joint-Stock Company"Dairy Plant"Stavropolsky"	Svetlana et al. 2024
RU2790676C1	Plant-based probiotic composition and method for its production	The composition, ranging from 1.5 to 6.5 pH, contains microelements, antioxidants, polyphenols, dietary fibres, peptides, beta-glucans, carbohydrates, proteins, fatty acids, active microorganisms, and postbiotics	Limited Liability Company"Probioducts"	Sergey et al. 2023
RU2658777C1	Method of postbiotic product production (options)	The invention group pertains to food and pharmaceutical industries, focusing on the production of postbiotic products, including fragments of lactic acid and probiotic microorganisms and their metabolic products	Andrey Vasilyevich Kazakov	Andrey et al. 2018
TW202142228A	Use of postbiotics extract to promote skin regeneration and anti-ageing capable of effectively promoting generation of human fibroblast and secreting anti-inflammatory hormone	Use of epibiotic extract to promote skin regeneration and anti-aging	Chuangbai Co., Ltd	Lin et al. 2021
TW202118498A	Composition and vaginal cleaning composition for inhibiting vaginal pathogens and uses thereof	Postbiotics, fermented from *L. salivarius, L. rhamnosus*, *Bifidobacterium animalis*, and *Streptococcus thermophiles*, are used in food, pharmaceutical, and vaginal cleaning compositions to inhibit vaginal pathogens	Fenghua Biotechnology Co., Ltd	Xie et al. 2022
US20230218505A1	Cosmetic use of engineered postbiotics comprising bacteriocins and/or endolysins	The method involves applying a postbiotic composition, comprising preferably a bacterial lysate and bacteriocin or endolysin, which work synergistically in the cosmetic caring method	Eligo Bioscience	Antoine And Xavier 2023
US11471433B1	Postbiotic compositions and related methods for agriculture	Methods for increasing insects’ fitness for agriculture or commerce by incorporating postbiotic agents like short chain fatty amino acids	Flagship Pioneering Innovations V Inc	Ignacio et al. 2022
US20230364166A1	Application of postbiotics of inactivated *Lactobacillus casei* I0B-P9 in blood glucose reducing	*L. casei* I0B-P9 strain exhibits high activity, and its postbiotics can effectively delay and control type II diabetes, making it suitable for diabetic products	Tianjin Innoorigin Biological Technology Co Ltd	Xuemei et al. 2023
US20220096573A1	Method for improving skin condition with postbiotic extract	Postbiotic extract improves skin condition by enhancing skin condition through isoelectric points, probiotic microorganism administration, pH adjustment, and cell wall isolation treatment for skin care and dermatological therapy	CHAMBIO Co Ltd. Chambio Co Ltd. Taiwan	Meei-Yn et al. 2022
US20240024378A1	Method and system for modulating an individual's skin microbiome	Skin contacting formulations containing beneficial bacteria, postbiotics, metabolites, and other microbe components are used to improve the health and maintenance of an individual's skin microbiome	Seed Health Inc	Sheri et al. 2024
US20230217979A1	Nutritional supplements and methods of nutritional supplementation affecting weight loss	Nutritional supplements, including probiotics like *Lactobacillus*, postbiotics like *Bifidobacterium*, prebiotics like fibre, and phytobiotics like plant extracts, are used to increase weight loss in subjects	Amare Global	Shawn 2023
US20240033299A1	Composition for a faecal-derived sterilised postbiotic in anti-ageing and neurotherapeutic applications	The study presents a method for analysing the anti-ageing effect of a sterilised postbiotic derived from faecal matter, utilising a baseline biomarker and a dosing schedule	Thaena Inc	Andrea et al. 2024
US10758577B2	Probiotics and fermentation metabolites for the prevention and treatment of disease conditions in animals	Compositions comprising a mixture of microbes, and the metabolites produced when the microbes are grown together	Pure Cultures 2020 Inc	Steven And Naseer 2020
US11452686B2	Cosmetic active substance obtained through bioconversion by *Lactobacillus arizonensis* of its original substrate, method for obtaining same composition comprising same, and uses	The invention pertains to a cosmetic active substance bioconverted by *L. arizonensis* from *Simmondisia chinens*, including its preparation method, compositions, and uses for cosmetic postbiotics applications	Societe Industrielle Limousine Application Biologique SA SILAB	Jean 2022

### Future perspective

Postbiotics, which are simply defined as non-viable microbial cells and their metabolic byproducts, have been noted to be potential alternatives to probiotics in human nutrition, animal feed, cosmetics, and pharmaceutical applications. These functional biotic products effectively bridge the gap between traditional probiotics and next-generation therapeutic approaches as they offer several advantages over the more popular probiotics, especially reduced risk of antibiotic resistance, easier processability, storage stability, and industrial scale-up. It has been noted that their stability and efficacy make them ideal candidates for incorporation into a wide range of products. However, the transition of postbiotics from the concept stage to industrial reality requires standardised definitions, methodologies, and rigorous scientific validation that will explicitly distinguish them from probiotics and other functional biotics. As highlighted in this paper, the processing route of postbiotics, such as the mode of inactivation, enzymatic treatment, and fermentation type, are critical factors concerning their production and variability in the conditions for these processes may lead to heterogeneity in postbiotic preparations, raising concerns about their inconsistency, both in research and in the industry. Thus, establishing standardised protocols for postbiotic processing will facilitate reproducibility and enhance regulatory approval for their real-time usage in food, pharmaceuticals, and other industries. Interestingly, their efficacy in ameliorating various health conditions such as gut microbiota imbalance, pathogenic infection, oxidative stress, and their various associated pathologies have now been established, and these have been attributed to their biochemical components, especially the peptides, polysaccharides, and organic acids. The major basis for these health benefits has been identified to be the propensity of these bioconstituents to interact with host cells, modulate gut microbiota, and influence systemic health. Nevertheless, understanding the structure-function relationships of these biological components is considered imperative in order to maximise their target-specific applications and optimise their formulation. Advances in omics approaches, such as transcriptomics, proteomics, and metabolomics also present promising avenues to reveal the complex mechanisms underlying postbiotic actions. Similarly, personalised nutrition and precision medicine offer interesting opportunities to tailor postbiotic interventions to individual needs. Although the current patent landscape and market trends demonstrate a significant interest in postbiotics-based products, a lot still has to be done with respect to regulatory clarity as well as standardised pre-clinical and clinical studies. In summary, postbiotics represent a transformative category in the spectrum of functional biotics, with significant implications for redefining approaches to health and nutrition as they meet the growing demand for safe, effective, and sustainable solutions. Continued research, innovation, and interdisciplinary collaboration will be instrumental in realising the full potential of postbiotics and shaping their future trajectory in functional foods and microbial biotherapy.

## Data Availability

No datasets were generated or analysed during the current study.

## References

[CR1] Abbasi A, Sabahi S, Bazzaz S, Tajani AG, Lahouty M, Aslani R, Hosseini H (2023) An edible coating utilizing Malva sylvestris seed polysaccharide mucilage and postbiotic from Saccharomyces cerevisiae var boulardii for the preservation of lamb meat. Int J Biol Macromol 246:125660. 10.1016/j.ijbiomac.2023.125660 37399877 10.1016/j.ijbiomac.2023.125660

[CR2] Abbasi A, Sheykhsaran E, Kafil HS (2021) Main methods for the preparation of postbiotics. In: Abbasi A, Sheykhsaran E, Kafil HS (eds) Postbiotics science technology and applications. Bentham Science Publishers, Sharjah, pp 38–47. 10.2174/9781681088389121010006

[CR3] Abdel Tawab FI, Abd Elkadr MH, Sultan AM, Hamed EO, El-ZayatAS AMN (2023) Probiotic potentials of lactic acid bacteria isolated from Egyptian fermented food. Sci Rep 13(1):1660. 10.1038/s41598-023-43752-0 37789063 10.1038/s41598-023-43752-0 PMC10547719

[CR4] Abdelazez A, Algarni AG, Al Jumayi E, Abdel-Motaal H, Meng H, XC, (2022) Postbiotic gamma-aminobutyric acid and camel milk intervention as innovative trends against hyperglycemia and hyperlipidemia in streptozotocin-induced C57BL/6J diabetic mice. Front Microbiol. 13:943930. 10.3389/fmicb.2022.943930 35898909 10.3389/fmicb.2022.943930 PMC9313471

[CR5] Abd-Elwahed ES, El-Waseif AA, Maany DA (2023) Biosynthesis and FPLC purification of antibacterial peptide from the biotherapeutic agent *Enterococcus faecium*. Egypt Pharm J 22(2):202–208. 10.4103/epj.epj_143_22

[CR6] Aghebati-Maleki L, Hasannezhad P, Abbasi A, Khani N (2021) Antibacterial, antiviral, antioxidant, and anticancer activities of postbiotics: a review of mechanisms and therapeutic perspectives. Biointerface Res Appl Chem 12(2):2629–2645. 10.33263/BRIAC122.26292645

[CR7] Aguilar-Toalá J, Garcia-Varela R, Garcia H, Mata-Haro V, González-Córdova A, Vallejo-Cordoba B, Hernández-Mendoza A (2018) Postbiotics: an evolving term within the functional foods field. Trend Food Sci Technol 75:105–114. 10.1016/j.tifs.2018.03.009

[CR8] Aguilar-Toalá J, Hall F, Urbizo-Reyes U, Garcia H, Vallejo-Cordoba B, González-Córdova A, Hernández-Mendoza A, Liceaga A (2020) In silico prediction and in vitro assessment of multifunctional properties of postbiotics obtained from two probiotic bacteria. Probiotics Antimicrob Prot 12:608–622. 10.1007/s12602-019-09568-z 10.1007/s12602-019-09568-z 31280464

[CR9] Ahmad Rather I, Seo BJ, Rejish Kumar VJ, Choi UH, Choi KH, Lim JH, Park YH (2013) Isolation and characterization of a proteinaceous antifungal compound from Lactobacillus plantarum YML007 and its application as a food preservative. Lett Appl Microbiol 57(1):69–76. 10.1111/lam.12077 23565693 10.1111/lam.12077

[CR10] Alan Y, Savcı A, Koçpınar EF, Ertaş M (2022) Postbiotic metabolites, antioxidant and anticancer activities of probiotic *Leuconostoc pseudomesenteroides* strains in natural pickles. Archiv Microbiol 204(9):571. 10.1007/s00203-022-03180-6 10.1007/s00203-022-03180-6 35997840

[CR11] Aliouche N, Sifour M, Ouled-Haddar H (2024) Exploring the antioxidant, antidiabetic, and antibacterial potential of postbiotic compounds derived from *Lactiplantibacillus plantarum* O7S1. Biotechnologia 105(3):215. 10.5114/bta.2024.141802 39439713 10.5114/bta.2024.141802 PMC11492891

[CR12] Almanaa TN, Rabie G, El-Mekkawy RM, Yassin MA, Saleh N, El-Gazzar N (2021) Antioxidant, antimicrobial and antiproliferative activities of fungal metabolite produced by *Aspergillus flavus* on in vitro study. Food Sci Technol 42:e01421. 10.1590/fst.01421

[CR13] Allied Market Research (2021) Postbiotic market size, share, competitive landscape and trend analysis report, by form, by application : global opportunity analysis and industry forecast, 2021 – 2031. https://www.alliedmarketresearch.com/postbiotic-market-A31027. Accessed 15 Sept 2024

[CR14] Amiri S, Rezazadeh-Bari M, Alizadeh-Khaledabad M, Rezaei-Mokarram R, Sowti-Khiabani M (2021) Fermentation optimization for co-production of postbiotics by *Bifidobacterium lactis* BB12 in cheese whey. Waste Biomass Valoris 12:5869–5884. 10.1007/s12649-021-01429-7

[CR15] Anand S, Kaur H, Mande SS (2016) Comparative in silico analysis of butyrate production pathways in gut commensals and pathogens. Front Microbiol 7:1945. 10.3389/fmicb.2016.01945 27994578 10.3389/fmicb.2016.01945 PMC5133246

[CR16] Andrea M, Piper D (2023) Composition of matter and methods for a fecal-derived sterilized prebiotic and postbiotic. Patent no: EP4045069A1

[CR17] Andrea M, Piper D, Brice T (2024) Composition for a fecal-derived sterilized postbiotic in anti-aging and neurotherapeutic applications. Patent no: US20230089315A1

[CR18] Andrey VK, Pavel AK, Ilya AK (2018) Method of postbiotic product production (options). Patent no: RU2658777C1.

[CR19] Angulo M, Reyes-Becerril M, Medina-CórdovaN T-R, Angulo C (2020) Probiotic and nutritional effects of *Debaryomyces hansenii* on animals. Appl Microbiol Biotechnol 104:7689–7699. 10.1007/s00253-020-10780-z 32686006 10.1007/s00253-020-10780-z

[CR20] Antoine D, Xavier D (2023) Cosmetic use of engineered postbiotics comprising bacteriocins and/or endolysins. Patent no: US20230218505A1.

[CR21] Arrioja-Bretón D, Mani-López E, Palou E, López-Malo A (2020) Antimicrobial activity and storage stability of cell-free supernatants from lactic acid bacteria and their applications with fresh beef. Food Control 115:107286. 10.1016/j.foodcont.2020.107286

[CR22] Avramia I, Amariei S (2022) A simple and efficient mechanical cell disruption method using glass beads to extract β-glucans from spent brewer’s yeast. Appl Sci 12(2):648. 10.3390/app12020648

[CR23] Azad MAK, Sarker MWD, Wan D (2018) Immunomodulatory effects of probiotics on cytokine profiles. BioMed Res Int 2018:8063647. 10.1155/2018/8063647 30426014 10.1155/2018/8063647 PMC6218795

[CR24] Balaguer F, Enrique M, Llopis S, Barrena M, Navarro V, Álvarez B, Chenoll E, Ramon D, Tortajada M, Martorell P (2022) Lipoteichoic acid from Bifidobacterium animalis subsp lactis BPL1: a novel postbiotic that reduces fat deposition via IGF-1 pathway. Microb Biotechnol 15(3):805–816. 10.1111/1751-7915.13769 33620143 10.1111/1751-7915.13769 PMC8913875

[CR25] Baumgardner RM, Berreta A, Kopper JJ (2021) Evaluation of commercial probiotics for antimicrobial resistance genes. Can Vet J 62(4):379–383 33867550 PMC7953935

[CR26] Beres C, Cabezudo I, Maidin NM (2020) Metabolites of polyphenols produced by probiotic microorganisms and their beneficial effects on human health and intestinal microbiota, In: Cavalcanti de Albuquerque MAC, Leblanc AM, Bedani R (ed) Lactic acid bacteria. CRC, Florida, pp 223-240. 10.1201/9780429422591

[CR27] Beteri B, Barone M, Turroni S, Brigidi P, Tzortzis G, Vulevic J, Sekulic K, Motei DE, Costabile A (2024) Impact of combined prebiotic galacto-oligosaccharides and *Bifidobacterium breve*-derived postbiotic on gut microbiota and HbA1c in prediabetic adults: a double-blind, randomized, placebo-controlled study. Nutrients 16(14):2205. 10.3390/nu16142205 39064648 10.3390/nu16142205 PMC11280236

[CR28] Bhatia L, Sarangi PK, Singh AK, Srivastava RK, Chandel AK (2024) Pre-, pro-, and postbiotics development from vegetable, fruit, and lignocellulosic biomass: a perspective. Food Biosci 104589. 10.1016/j.fbio.2024.104589

[CR29] Blazheva D, Mihaylova D, Averina O, Slavchev A, Brazkova M, Poluektova E, Danilenko V, Krastanov A (2022) Antioxidant potential of probiotics and postbiotics: a biotechnological approach to improving their stability. Russ J Genet 58(9):1036–1050. 10.1134/S1022795422090058

[CR30] Cai Y, Yuan W, Wang S, Guo W, Li A, Wu Y, Chen X, Ren Z, Zhou Y (2019) In vitro screening of putative probiotics and their dual beneficial effects: to white shrimp (*Litopenaeus vannamei*) postlarvae and to the rearing water. Aquaculture 498:61–71. 10.1016/j.aquaculture.2018.08.024

[CR31] Chaney E, Miller EA, Firman J, Binnebose A, Kuttappan V, Johnson TJ (2023) Effects of a postbiotic, with and without a saponin-based product, on turkey performance. Poult Sci 102(5):102607. 10.1016/j.psj.2023.102607 36933527 10.1016/j.psj.2023.102607 PMC10036732

[CR32] Chaudhary A, Prajapati N, Prajapati A, Singh S, Joshi M, Prajapati D, Patani A, Sahoo DK, Patel A (2024) Postbiotic emissaries: a comprehensive review on the bioprospecting and production of bioactive compounds by *Enterococcus* species. Int J Food Sci Technol 59(10):6769–6782. 10.1111/ijfs.17431

[CR33] Chen L, Ning C, Ma L, Wang M, Zhao Y, Li S, Zhao Z (2023) Harnessing the potential of postbiotics derived from *Bacillus coagulans*: a promising avenue for obesity management via the gut-liver axis. Food Biosci 56:103108. 10.1016/j.fbio.2023.103108

[CR34] Cho H, Junga K, Song SS (2021) A composition for preventing or relieving hangover of the comprising heat-killed *Lactobacillus salivarius* v133 as an active ingredient. Patent no: KR102251295B1.

[CR35] Chuah LO, Foo HL, Loh TC, Mohammed Alitheen NB, Yeap SK, Abdul Mutalib NE, Abdul Rahim R, Yusoff K (2019) Postbiotic metabolites produced by *Lactobacillus* plantarum strains exert selective cytotoxicity effects on cancer cells. BMC Complement Alt Med 19:1–12. 10.1186/s12906-019-2528-2 10.1186/s12906-019-2528-2 PMC6547513 31159791

[CR36] Chung HJ, Lee H, Kim M, Lee JW, Saeed M, Lee H, Jung SH, Shim JJ, Lee JL, Heo K (2022) Development and metabolic profiling of a postbiotic complex exhibiting antibacterial activity against skin microorganisms and anti-inflammatory effect on human keratinocytes. Food Sci Biotechnol 31(10):1325–1334. 10.1007/s10068-022-01123-x 35992320 10.1007/s10068-022-01123-x PMC9385932

[CR37] Cui S, Mao B, Zhang Q, Zhao J (2023) Post-growth hormone prepared from *Lactobacillus paracasei* and having effects of promoting host HA synthesis and enhancing HA application. Patent no: CN115927122A.

[CR38] Dai D, Kong F, Han H, Shi W, Song H, Yoon I, Wang S, Liu X, Lu N, Wang W, Li S (2024) Effects of postbiotic products from *Saccharomyces cerevisiae* fermentation on lactation performance, antioxidant capacities, and blood immunity in transition dairy cows. J Dairy Sci 107(12):10584–10598. 10.3168/jds.2023-24435 39004128 10.3168/jds.2023-24435

[CR39] Dameshghian M, Tafvizi F, Tajabadi Ebrahimi M, Hosseini Doust R (2024) Anticancer potential of postbiotic derived from *Lactobacillus brevis* and *Lactobacillus casei*: in vitro analysis of breast cancer cell line. Probiotics Antimicrob Prot 1–14. 10.1007/s12602-024-10288-2 10.1007/s12602-024-10288-2 38758482

[CR40] da Silva SS, Pérez-Rodríguez N, Domínguez J, de Souza OR (2017) Inhibitory substances production by Lactobacillus plantarum ST16Pa cultured in hydrolysed cheese whey supplemented with soybean flour and their antimicrobial efficiency as biopreservatives on fresh chicken meat. Food Res Int 99:762–769. 10.1016/j.foodres.2017.05.026 28784542 10.1016/j.foodres.2017.05.026

[CR41] Datta S, Timson DJ, Annapure US (2017) Antioxidant properties and global metabolite screening of the probiotic yeast Saccharomyces cerevisiae var. boulardii. J Sci Food Agric 97(9):3039–3049. 10.1002/jsfa.8147 27868205 10.1002/jsfa.8147

[CR42] Deng F, Chen Y, Sun T, Wu Y, Su Y, Liu C, Zhou J, Deng Y, Wen J (2021) Antimicrobial resistance, virulence characteristics and genotypes of Bacillus spp from probiotic products of diverse origins. Food Res Int 139:109949. 10.1016/j.foodres.2020.109949 33509502 10.1016/j.foodres.2020.109949

[CR43] Do H, Li ZR, Tripathi PK, Mitra S, Guerra S, Dash A, Weerasekera D, Makthal N, Shams S, Aggarwal S (2024) Engineered probiotic overcomes pathogen defences using signal interference and antibiotic production to treat infection in mice. Nat Microbiol 9(2):502–513. 10.1038/s41564-023-01583-9 38228859 10.1038/s41564-023-01583-9 PMC10847043

[CR44] dos Santos FA, Barroso FA, Campos GM, Américo MF, dos Santos Viegas RC, Gomes GC, Vital KD, Fernandes SO, de Oliveira Carvalho RD, Jardin J (2024) Exploring the anti-inflammatory effects of postbiotic proteins from *Lactobacillus delbrueckii* CIDCA 133 on inflammatory bowel disease model. Int J Biol Macromol 277:134216. 10.1016/j.ijbiomac.2024.134216 39069058 10.1016/j.ijbiomac.2024.134216

[CR45] Ebrahimi M, Sadeghi A, Rahimi D, Purabdolah H, Shahryari S (2021) Postbiotic and anti-aflatoxigenic capabilities of *Lactobacillus**kunkeei* as the potential probiotic LAB isolated from the natural honey. Probiotics Antimicrob Prot 13:343–355. 10.1007/s12602-020-09697-w 10.1007/s12602-020-09697-w 32803518

[CR46] Eli R, Edwards G, John NT, Antony SR, Sandesh ST (2023) Formulation for the treatment of inflammatory bowel diseases using whole plant fiber extract from sugarcane. Patent no: JP7385930B2.

[CR47] Erfanian N, Safarpour H, Tavakoli T, Mahdiabadi MA, Nasseri S, Namaei MH (2024) Investigating the therapeutic potential of *Bifidobacterium breve* and *Lactobacillus rhamnosus* postbiotics through apoptosis induction in colorectal HT-29 cancer cells. Iran J Microbiol 16(1):68–78 38682058 10.18502/ijm.v16i1.14873 PMC11055435

[CR48] Eze UJ, Lal A, Elkoush MI, Halytska M, Atif S (2024) Recurrent *Lactobacillus rhamnoses* bacteremia and complications in an immunocompromised patient with history of probiotic use: a case report. Cureus 16(2):e54879–e54879. 10.7759/cureus.54879 38550408 10.7759/cureus.54879 PMC10976466

[CR49] Fang F, LiY LuX, Wu K, Zhou L, Sun Y, Wu J, Gao J (2023) Effect of potential postbiotics derived from food-isolated *Lactobacillus parabuchneri* on different enterotypes of human gut microbiome. LWT 182:114782. 10.1016/j.lwt.2023.114782

[CR50] Fang Z, Bhandari B (2017) Spray drying of bioactives. In: Roos Y, Livney Y (eds) Engineering foods for bioactives stability and delivery. Food engineering series. Springer, New York, pp 261–284. 10.1007/978-1-4939-6595-3

[CR51] Feito J, Araujo C, Arbulu S, Contente D, Gómez-Sala B, Díaz-Formoso L, Muñoz-Atienza E, Borrero J, Cintas LM, Hernández PE (2023) Design of *Lactococcus lactis* strains producing garvicin A and/or Garvicin Q, either alone or together with nisin A or Nisin Z and high antimicrobial activity against *Lactococcus garvieae*. Foods 12(5):1063. 10.3390/foods12051063 36900581 10.3390/foods12051063 PMC10000435

[CR52] Feye K, Carroll J, Anderson K, Whittaker J, Schmidt-McCormack G, McIntyre D, Pavlidis H, Carlson S (2019) Saccharomyces cerevisiae fermentation products that mitigate foodborne Salmonella in cattle and poultry. Front Vet Sci 6:107. 10.3389/fvets.2019.00107 31024942 10.3389/fvets.2019.00107 PMC6467977

[CR53] Frappier M, Auclair J, Bouasker S, Gunaratnam S, Diarra C, Millette M (2022) Screening and characterization of some *Lactobacillaceae* for detection of cholesterol-lowering activities. Probiotics Antimicrob Prot 14(5):873–883. 10.1007/s12602-022-09959-9 10.1007/s12602-022-09959-9 PMC9474388 35704269

[CR54] Fu J, Liu J, Wen X, Zhang G, Cai J, Qiao Z, An Z, Zheng J, Li L (2023) Unique probiotic properties and bioactive metabolites of *Saccharomyces boulardii*. Probiotics Antimicrob Prot 15(4):967–982. 10.1007/s12602-022-09953-1 10.1007/s12602-022-09953-1 35608794

[CR55] Future Market Insights (2024) Postbiotic supplements market outlook for 2024 to 2034. https://www.futuremarketinsights.com/reports/postbiotic-supplements-market. Accessed 15 Sept 2024

[CR56] Gangaraju D, Raghu AV, Siddalingaiya Gurudutt P (2022) Green synthesis of γ-aminobutyric acid using permeabilized probiotic *Enterococcus faecium* for biocatalytic application. Nano Sel 3(10):1436–1447. 10.1002/nano.202200059

[CR57] Gezginç Y, Karabekmez-erdem T, Tatar HD, Ayman S, Ganiyusufoğlu E, Dayısoylu KS (2022) Health promoting benefits of postbiotics produced by lactic acid bacteria: exopolysaccharide. Biotech Stud 31(2):61–70. 10.38042/biotechstudies.1159166

[CR58] Gill P, Staudacher HM (2023) Are postbiotics key to the potential benefits of fermented foods? Lancet Gastroenterol Hepatol 8(6):509. 10.1016/s2468-1253(23)00120-6

[CR59] Gingold-Belfer R, Levy S, Layfer O, Pakanaev L, Niv Y, Dickman R, Perets TT (2020) Use of a novel probiotic formulation to alleviate lactose intolerance symptoms-A pilot study. Probiotics & Antimicrob Prot 12:112–118. 10.1007/s12602-018-9507-7 10.1007/s12602-018-9507-7 30617948

[CR60] Gomes TA, Zanette CM, Spier MR (2020) An overview of cell disruption methods for intracellular biomolecules recovery. Prep Biochem Biotechnol 50(7):635–654. 10.1080/10826068.2020.1728696 32074000 10.1080/10826068.2020.1728696

[CR61] Grigorov E, Kirov B, Marinov MB, Galabov V (2021) Review of microfluidic methods for cellular lysis. Micromach 12(5):498. 10.3390/mi12050498 10.3390/mi12050498 PMC8145176 33925101

[CR62] Gu X, Wang H, Wang L, Zhang K, Tian Y, Wang X, Xu G, Guo Z, Ahmad S, Egide H (2024) The Antioxidant Activity and Metabolomic Analysis of the Supernatant of Streptococcus Alactolyticus Strain FGM Sci Rep 14(1):8413. 10.1038/s41598-024-58933-8 38600137 10.1038/s41598-024-58933-8 PMC11006861

[CR63] Guardia, AM (2024) Postbiotic food additive for products obtained from cereal flour, food composition and food product comprising same. Igen Biolab Group AG Patent no: FR3133727B3

[CR64] Gueniche A, Liboutet M, Cheilian S, Fagot D, Juchaux F, Breton L (2021) Vitreoscilla filiformis extract for topical skin care: a review. Front Cell Infect Microbiol 11:747663. 10.3389/fcimb.2021.747663 34976852 10.3389/fcimb.2021.747663 PMC8717924

[CR65] Guérin M, Silva CR, Garcia C, Remize F (2020) Lactic acid bacterial production of exopolysaccharides from fruit and vegetables and associated benefits. Fermentation 6(4):115. 10.3390/fermentation6040115

[CR66] Hamad G, Abdelmotilib N, Darwish AM, Zeitoun A (2020) Commercial probiotic cell-free supernatants for inhibition of Clostridium perfringens poultry meat infection in Egypt. Anaerobe 62:102181. 10.1016/j.anaerobe.2020.102181 32092413 10.1016/j.anaerobe.2020.102181

[CR67] Han SD, Youngsun H, Minju P, Cho HI, Eunseok L (2022) *Lactobacillus* paracasei EPS DA-BACS promoting the growth of *Bifidobacterium* and inhibiting *Clostridium difficile* and polysaccharide therefrom. Patent no: KR102374480B1

[CR68] Heniedy A, Mahdy D, Elenien WIA, Mourad S, El-Kadi R (2024) Postbiotics as a health-promoting technique: a review article on scientific and commercial interest. Process Biochem 144:6–19. 10.1016/j.procbio.2024.05.010

[CR69] Hijová E (2024) Postbiotics as metabolites and their biotherapeutic potential. Int J Mol Sci 25(10):5441. 10.3390/ijms25105441 38791478 10.3390/ijms25105441 PMC11121590

[CR70] Hossain MI, Mizan MF, Roy PK, Nahar S, Toushik SH, Ashrafudoulla M, Jahid IK, Lee J, Ha SD (2021) Listeria monocytogenes biofilm inhibition on food contact surfaces by application of postbiotics from Lactobacillus curvatus B. 67 and Lactobacillus plantarum M. 2. Food Res Int. 148:110595. 10.1016/j.foodres.2021.110595 34507740 10.1016/j.foodres.2021.110595

[CR71] Hu J, Lin Y, Zhang Z, Xiang T, Mei Y, Zhao S, Liang Y, Peng N (2016) High-titer lactic acid production by *Lactobacillus pentosus* FL0421 from corn stover using fed-batch simultaneous saccharification and fermentation. Biores Technol 214:74–80. 10.1016/j.biortech.2016.04.034 10.1016/j.biortech.2016.04.034 27128191

[CR72] Huang Y, Zhao S, Yao K, Liu D, Peng X, Huang J, Huang Y, Li L (2020) Physicochemical, microbiological, rheological, and sensory properties of yoghurts with new polysaccharide extracts from Lactarius volemus Fr using three probiotics. Int J Dairy Technol 73(1):168–181. 10.1111/1471-0307.12653

[CR73] Ibraheim HK, Madhi KS, Baqer GK, Gharban HA (2023) Effectiveness of raw bacteriocin produced from lactic acid bacteria on biofilm of methicillin-resistant Staphylococcus aureus. Vet World 16(3):491–499. 10.14202/vetworld.2023.491-499 37041833 10.14202/vetworld.2023.491-499 PMC10082751

[CR74] Ignacio M, Maier, SA, Thomas MM, Emily MM (2022) Postbiotic compositions and related methods for agriculture. Patent no: US11471433B1.

[CR75] İncili GK, Karatepe P, Akgöl M, Kaya B, Kanmaz H, Hayaloğlu AA (2021) Characterization of *Pediococcus acidilactici* postbiotic and impact of postbiotic-fortified chitosan coating on the microbial and chemical quality of chicken breast fillets. Int J Biol Macromol 184:429–437. 10.1016/j.ijbiomac.2021.06.106 34166693 10.1016/j.ijbiomac.2021.06.106

[CR76] Isaac-Bamgboye FJ, Mgbechidinma CL, Onyeaka H, Isaac-Bamgboye IT, Chukwugozie DC (2024) Exploring the potential of postbiotics for food safety and human health improvement. J Nutr Metabol 2024(1):186816. 10.1155/2024/1868161 10.1155/2024/1868161 PMC11321893 39139215

[CR77] Ishikawa K, Bueno M, Kawamoto D, Simionato M, Mayer M (2021) Lactobacilli postbiotics reduce biofilm formation and alter transcription of virulence genes of Aggregatibacter actinomycetemcomitans. Mol Oral Microbiol 36(1):92–102. 10.1111/omi.12330 33372378 10.1111/omi.12330

[CR78] Izuddin WI, Humam AM, Loh TC, Foo HL, Samsudin AA (2020) Dietary postbiotic *Lactobacillus plantarum* improves serum and ruminal antioxidant activity and upregulates hepatic antioxidant enzymes and ruminal barrier function in post-weaning lambs. Antioxidants 9(3):250. 10.3390/antiox9030250 32204511 10.3390/antiox9030250 PMC7139658

[CR79] Jae GS, Joo HS, Do KL (2023) Novel *Ficalibacterium prausnitzii* strain EB-FPDK11 and its uses. Patent no: JP7395622B2

[CR80] Jae KC, Jung K, Rinkai S, Wonjong L, Dongho S, Lee DH (2023) Makgeolli-derived *Lacticaseibacillus paracasei* subsp. paracasei DCF0429 and functional postbiotics using the same. Patent no: KR102542030B1

[CR81] Jaegyu L, Lee DS, Lee D, Minhyuk C, Yisuan Yoonjung K (2022) Composition comprising complex enzymes to improve digestion and toxin excretion. Patent no: KR102427841B1

[CR82] Jansson-Knodell CL, Krajicek EJ, Ramakrishnan M, Rogers NA, Siwiec R, Bohm M, Nowak T, Wo J, Lockett C, Xu H (2022) Relationships of intestinal lactase and the small intestinal microbiome with symptoms of lactose intolerance and intake in adults. Dig Dis Sci 67(12):5617–5627 35322314 10.1007/s10620-022-07469-w PMC11075758

[CR83] Jasim SA, Abdelbasset WK, Shichiyakh RA, Al-Shawi SG, Yasin G, Jalil AT, Karim YS, Mustafa YF, Norbakhsh M (2022) Probiotic effects of the fungi*, Aspergillus niger* on growth, immunity, haematology, intestine fungal load and digestive enzymes of the common carp. Cyprinus Carpio Aquacult Res 53(10):3828–3840. 10.1111/are.15890

[CR84] Jean P (2022) Cosmetic active substance obtained through bioconversion by Lactobacillus arizonensis of its original substrate, method for obtaining same composition comprising same, and uses. Patent no: US11452686B2.

[CR85] Jeongju L, Jung CB (2023). Washing machine cleaner composition comprising postbiotics component and washing machine cleaner container taking the washing machine cleaner composition comprising postbiotics component. Patent no: KR102513864B1

[CR86] Jiang Y, Tommy Li Y, Yang X (2023). Composition containing *Lactobacillus rhamnosus* JL1 metagenesis powder, preparation method and application. Patent no: CN115414392B.

[CR87] Joella B, Richard SC, Michael SS (2019) Animal feed compositions and feed additives. Patent no: AU2015296538B2

[CR88] Johnson CN, Kogut MH, Genovese K, He H, Kazemi S, Arsenault RJ (2019) Administration of a postbiotic causes immunomodulatory responses in broiler gut and reduces disease pathogenesis following challenge. Microorganisms 7(8):268. 10.3390/microorganisms7080268 31426502 10.3390/microorganisms7080268 PMC6723925

[CR89] Jung Y, Kim HS, Jaygal G, Cho HR, Bae Lee K, Song IB, Kim JH, Kwak MS, Han KH, Bae MJ (2022) Postbiotics enhance NK cell activation in stress-induced mice through gut microbiome regulation. J Microbiol Biotechnol 32(5):612–620. 10.4014/jmb.2111.11027 35283424 10.4014/jmb.2111.11027 PMC9628878

[CR90] Jung MS, Jae HL (2024) Composition containing polysaccharide or extract derived from *Lactobacillus plantarum*. Patent no: JP2024510374A

[CR91] Justge Ma Q, Yu G (2022) Application of *Acermanium* and *Acermanium* postbiotic in preparation of medicine or health-care product for preventing or treating hepatic steatosis. Patent no: CN115463159A

[CR92] Kang CH, Kim JS, Park HM, Kim S, Paek NS (2021) Antioxidant activity and short-chain fatty acid production of lactic acid bacteria isolated from Korean individuals and fermented foods. 3 Biotech 11(5):217. 10.1007/s13205-021-02767-y 33936926 10.1007/s13205-021-02767-y PMC8050147

[CR93] Kareem KY, Hooi Ling F, Teck Chwen L, May Foong O, Anjas Asmara S (2014) Inhibitory activity of postbiotic produced by strains of *Lactobacillus plantarum* using reconstituted media supplemented with inulin. Gut Pathog 6(23):1–7. 10.1186/1757-4749-6-23 24991236 10.1186/1757-4749-6-23 PMC4076511

[CR94] Karuvelan M, Sumukhi SS, Rajakannu S, Chelliah R, Barathikannan K, Vijayalakshmi S, Rubab M, Oh DH (2025) Immunomodulatory and growth-enhancing postbiotic effects in poultry. In: Dharumadurai D, Halami PM (eds) Postbiotics. Academic Press, New York, pp 575–587. 10.1016/B978-0-443-22188-0.00033-4

[CR95] Kathayat D, Closs G Jr, Helmy YA, Lokesh D, Ranjit S, Rajashekara G (2021) Peptides affecting the outer membrane lipid asymmetry system (MlaA-OmpC/F) reduce avian pathogenic *Escherichia**coli* (APEC) colonization in chickens. Appl Environ Microbiol 87(17):e00567-e521. 10.1128/AEM.00567-21 34132592 10.1128/AEM.00567-21 PMC8357279

[CR96] Kerksick CM, Moon JM, Jäger R (2024) It’s dead! can postbiotics really help performance and recovery? a systematic review. Nutr 16(5):720. 10.3390/nu16050720 10.3390/nu16050720 PMC10933997 38474848

[CR97] Khani N, Abedi Soleimani R, Chadorshabi S, Moutab B, Milani P, Rad A (2024) Postbiotics as candidates in biofilm inhibition in food industries. Lett Appl Microbiol 77(4):069. 10.1093/lambio/ovad069 10.1093/lambio/ovad069 37309029

[CR98] Kim YS, Shin HY, Kim H, JeongEJ KHG, Suh MG, Suh HJ, Yu KW (2023) Anti-inflammatory active polysaccharide from postbiotics of *Cordyceps militaris* mycelium-liquid culture. Korean J Food Nutr 36(1):6–16. 10.9799/ksfan.2023.36.1.006

[CR99] Kim HJ, Youn HY, Moon JS, Kim H, Seo KH (2024a) Comparative anti-microbial and anti-biofilm activities of postbiotics derived from kefir and normal raw milk lactic acid bacteria against bovine mastitis pathogens. LWT 191:115699. 10.1016/j.lwt.2023.115699

[CR100] Kim JH, Kwak W, Nam Y, Baek J, Lee Y, Yoon S, Kim W (2024b) Effect of postbiotic Lactiplantibacillus plantarum LRCC5314 supplemented in powdered milk on type 2 diabetes in mice. J Dairy Sci 107(8):5301–5315. 10.3168/jds.2023-24103 38554828 10.3168/jds.2023-24103

[CR101] Knob A, Fortkamp D, Prolo T, Izidoro SC, Almeida JM (2014) Agro-residues as alternative for xylanase production by filamentous fungi. Bio Res 9(3):5738–5773. 10.15376/biores.9.3.5738-5773

[CR102] Krawczyk R, Banaszkiewicz A (2021) Dr Józef Brudziński–the true ‘Father of probiotics.’ Benef Microb 12(3):211–213. 10.3920/BM2020.0201 10.3920/BM2020.0201 34057052

[CR103] Lalani AR, Rastegar-Pouyani N, Askari A, Tavajohi S, Akbari S, Jafarzadeh E (2024) Food Additives, Benefits, and Side Effects: A Review Article. J Chem. Health Risks 14(1). 10.22034/jchr.2023.1967340.1619

[CR104] Latif A, ShehzadA NS, Zahid A, Ashraf W, Iqbal MW, Rehman A, Riaz T, Aadil RM, Khan IM (2023) Probiotics: Mechanism of action, health benefits and their application in food industries. Front Microbiol 14:1216674. 10.3389/fmicb.2023.1216674 37664108 10.3389/fmicb.2023.1216674 PMC10470842

[CR105] Lee NK, Hong JY, Yi SH, Hong SP, Lee JE, Paik HD (2019a) Bioactive compounds of probiotic *Saccharomyces cerevisiae* strains isolated from cucumber jangajji. J Funct Foods 58:324–329. 10.1016/j.jff.2019.04.059

[CR106] Lee SY, Chew KW, Show PL (2019b) Cell separation and disruption, product recovery, and purification. In: Berenjian A (ed) Essentials in fermentation technology. Learning materials in biosciences. Springer, Cham, pp 237–271. 10.1007/978-3-030-16230-6_8

[CR107] Lee YS, Lee SJ, Jang WJ, Lee EW (2024) Protective effects of the postbiotic *Levilactobacillus brevis* BK3 against H2O2-Induced Oxidative Damage in Skin Cells. J Microbiol Biotechnol 34(7):1401–1409. 10.4014/jmb.2403.03010 38881180 10.4014/jmb.2403.03010 PMC11294649

[CR108] Lee SU (2022) An eco-friendly laundry detergent composition containing a post-biotic component and a laundry detergent composition manufacturing device for manufacturing an eco-friendly laundry detergent composition containing the post-biotic component. Patent no: KR102463565B1

[CR109] Liang B, Xing D (2023) The current and future perspectives of postbiotics. Probiotics Antimicrob Proteins 15(6):1626–1643. 10.1007/s12602-023-10045-x 36763279 10.1007/s12602-023-10045-x PMC9913028

[CR110] Li Z, Yi CX, Katiraei S, Kooijman S, Zhou E, Chung CK, Gao Y, van den Heuvel JK, Meijer OC, Berbée JF (2018) Butyrate reduces appetite and activates brown adipose tissue via the gut-brain neural circuit. Gut 67(7):1269–1279. 10.1136/gutjnl-2017-314050 29101261 10.1136/gutjnl-2017-314050

[CR111] Lin, M., Qiu, H., Qiu, Y (2021). Use of postbiotics extract to promote skin regeneration and anti-aging capable of effectively promoting generation of human fibroblast and secreting anti-inflammatory hormone. Patent no: TW202142228A.

[CR112] Lin S, Yang X, Long Y, Zhong H, Wang P, Yuan P, Zhang X, Che L, Feng B, Li J (2020) Dietary supplementation with Lactobacillus plantarum modified gut microbiota, bile acid profile and glucose homoeostasis in weaning piglets. Brit J Nutr 124(8):797–808. 10.1017/S0007114520001774 32436488 10.1017/S0007114520001774

[CR113] Ling H, Ling Z, Jia S, Qiu Y (2023) Preparation method and application of LGG fermentation product containing immunoregulatory peptide functional component. Patent no: CN111802649B

[CR114] Liu Y, Liu G, Fang J (2024) Progress on the mechanisms of *Lactobacillus plantarum* to improve intestinal barrier function in ulcerative colitis. J Nutr Biochem 124:109505. 10.1016/j.jnutbio.2023.109505 37890709 10.1016/j.jnutbio.2023.109505

[CR115] Ma X, Ren D, Yu Y, Yu X (2024) *Lactobacillus plantarum* postbiotic and use thereof in products for changing intestinal microecology and reducing cholesterol. Patent no: GB2620891A

[CR116] Machado A, Marinheiro L, Benson H, Grice J, Martins T, Lan A, Lopes P, Andreo-Filho N, Leite-Silva V (2023) A novel handrub tablet loaded with pre-and post-biotic solid lipid nanoparticles combining virucidal activity and maintenance of the skin barrier and microbiome. Pharmaceutics 15(12):2793. 10.3390/pharmaceutics15122793 38140133 10.3390/pharmaceutics15122793 PMC10747770

[CR117] Majeed M, Majeed S, Nagabhushanam K, Mundkur L, Rajalakshmi H, Shah K, Beede K (2020) Novel topical application of a postbiotic, LactoSporin®, in mild to moderate acne: a randomized, comparative clinical study to evaluate its efficacy, tolerability and safety. Cosmetics 7(3):70. 10.3390/cosmetics7030070

[CR118] Makino S, Sato A, Goto A, Nakamura M, Ogawa M, Chiba Y, Hemmi J, Kano H, Takeda K, Okumura K, Asami Y (2016) Enhanced natural killer cell activation by exopolysaccharides derived from yogurt fermented with Lactobacillus delbrueckii ssp. bulgaricus OLL1073R-1. J Dairy Sci 99(2):915–923. 10.3168/jds.2015-10376 26686726 10.3168/jds.2015-10376

[CR119] Markowiak-Kopeć P, Śliżewska K (2020) The effect of probiotics on the production of short-chain fatty acids by human intestinal microbiome. Nutrients 12(4):1107. 10.3390/nu12041107 32316181 10.3390/nu12041107 PMC7230973

[CR120] Martorell P, Alvarez B, Llopis S, Navarro V, Ortiz P, Gonzalez N, Balaguer F, Rojas A, Chenoll E, Ramon D (2021) Heat-treated *Bifidobacterium longum* CECT-7347: a whole-cell postbiotic with antioxidant, anti-inflammatory, and gut-barrier protection properties. Antioxidants 10(4):536. 10.3390/antiox10040536 33808122 10.3390/antiox10040536 PMC8067082

[CR121] Mathew S, Aronsson A, Karlsson EN, Adlercreutz P (2018) Xylo-and arabinoxylooligosaccharides from wheat bran by endoxylanases, utilisation by probiotic bacteria, and structural studies of the enzymes. Appl Microbiol Biotechnol 102:3105–3120. 10.1007/s00253-018-8823-x 29445853 10.1007/s00253-018-8823-x

[CR122] Mathur H, Linehan K, Flynn J, Byrne N, Dillon P, Conneely M, Grimaud G, Hill C, Stanton C, Ross RP (2022) Emulsion-based postbiotic formulation is comparable to viable cells in eliciting a localized immune response in dairy cows with chronic mastitis. Front Microbiol 13:759649. 10.3389/fmicb.2022.759649 35391729 10.3389/fmicb.2022.759649 PMC8981918

[CR123] Meei-Yn L, Hung C, Yi-Heng C (2022) Method for improving skin condition with postbiotic extract. Patent no: US20220096573A1

[CR124] Mehta JP, Ayakar S, Singhal RS (2023) The potential of paraprobiotics and postbiotics to modulate the immune system: a Review. Microbiol Res 275:127449. 10.1016/j.micres.2023.127449 37454427 10.1016/j.micres.2023.127449

[CR125] Minkyu Y, Lee CW, Doosung K, Jung JH, Kyungmin K, Seojin Y, Seunghoon L (2021) Manufacturing method for aminobiotics using medium comprising A2 beta casein, and amniobiotics prepared therethrough. Patent no: KR102332021B.

[CR126] Mishra B, Mishra AK, Mohanta YK, Yadavalli R, Agrawal DC, Reddy HP, Gorrepati R, Reddy CN, Mandal SK, Shamim MZ (2024a) Postbiotics: the new horizons of microbial functional bioactive compounds in food preservation and security. Food Prod Proc Nutr 6(1):28. 10.1186/s43014-023-00200-w

[CR127] Mishra N, Garg A, Ashique S, Bhatt S (2024b) Potential of postbiotics for the treatment of metabolic disorders. Drug Discov. Today: 103921. 10.1016/j.drudis.2024.103921 10.1016/j.drudis.2024.103921 38382867

[CR128] Mohammed S, Çon AH (2024) Postbiotic nanoparticles (postbiotics-NPs): a novel strategy for providing probiotics’ health advantages through food consumption. Food Sci. Biotechnol: 1–8. 10.1007/s10068-024-01629-6 10.1007/s10068-024-01629-6 PMC11339192 39184983

[CR129] Monica AC(2023) Quorum-sensing inhibitors and/or postbiotic metabolites and related methods. Patent no: EP3993819A1

[CR130] Moradi M, Mardani K, Tajik H (2019) Characterization and application of postbiotics of *Lactobacillus* spp. on Listeria monocytogenes in vitro and in food models. LWT 111:457–464. 10.1016/j.lwt.2019.05.072

[CR131] Moradi M, Kousheh SA, Almasi H, Alizadeh A, Guimarães JT, Yılmaz N, Lotfi A (2020) Postbiotics produced by lactic acid bacteria: the next frontier in food safety. Comp Rev Food Sci Food Saf 19(6):3390–3415. 10.1111/1541-4337.12613 10.1111/1541-4337.12613 33337065

[CR132] Motei DE, Beteri B, Hepsomali P, Tzortzis G, Vulevic J, Costabile A (2023) Supplementation with postbiotic from *Bifidobacterium breve* BB091109 improves inflammatory status and endocrine function in healthy females: a randomized, double-blind, placebo-controlled, parallel-groups study. Front Microbiol 14:1273861. 10.3389/fmicb.2023.1273861 38075921 10.3389/fmicb.2023.1273861 PMC10702524

[CR133] Motevaseli E, Dianatpour A, Ghafouri-Fard S (2017) The role of probiotics in cancer treatment: emphasis on their in vivo and in vitro anti-metastatic effects. Int. J. Mol. Cell. Med 6(2): 66. 10.22088/acadpub.BUMS.6.2.1 10.22088/acadpub.BUMS.6.2.1 PMC5581548 28890883

[CR134] Mukherjee A, Breselge S, Dimidi E, Marco ML, Cotter PD (2024) Fermented foods and gastrointestinal health: underlying mechanisms. Nat Rev Gastroenterol Hepatol 21(4):248–266. 10.1038/s41575-023-00869-x 38081933 10.1038/s41575-023-00869-x

[CR135] Nag D, Goel A, Padwad Y, Singh D (2023) In vitro characterisation revealed Himalayan dairy Kluyveromyces marxianus PCH397 as potential probiotic with therapeutic properties. Probiotic Antimicrob Prot 15(3):761–773. 10.1007/s12602-021-09874-5 10.1007/s12602-021-09874-5 35040023

[CR136] Nam Y, Kim J, Baek J, Kim W (2021) Improvement of cutaneous wound healing via topical application of heat-killed *Lactococcus chungangensis* CAU 1447 on diabetic mice. Nutrients 13(8):2666. 10.3390/nu13082666 34444827 10.3390/nu13082666 PMC8401197

[CR137] Nataraj BH, Ali SA, Behare PV, Yadav H (2020) Postbiotics-parabiotics: the new horizons in microbial biotherapy and functional foods. Microb Cell Fact 19:1–22. 10.1186/s12934-020-01426-w 32819443 10.1186/s12934-020-01426-w PMC7441679

[CR138] Nealon NJ, Worcester CR, Boyer SM, Haberecht HB, Ryan EP (2024) Metabolite profiling and bioactivity guided fractionation of *Lactobacillaceae* and rice bran postbiotics for antimicrobial-resistant *Salmonella**typhimurium* growth suppression. Front Microbiol 15:1362266. 10.3389/fmicb.2024.1362266 38659978 10.3389/fmicb.2024.1362266 PMC11040457

[CR139] Noh SY, Kang SS, Yun CH, Han SH (2015) Lipoteichoic acid from Lactobacillus plantarum inhibits Pam2CSK4-induced IL-8 production in human intestinal epithelial cells. Mol Immunol 64(1):183–189. 10.1016/j.molimm.2014.11.014 25481370 10.1016/j.molimm.2014.11.014

[CR140] Nowak A, Zakłos-Szyda M, Rosicka-Kaczmarek J, Motyl I (2022) Anticancer potential of post-fermentation media and cell extracts of probiotic strains: an in vitro study. Cancers 14(7):1853. 10.3390/cancers14071853 35406625 10.3390/cancers14071853 PMC8998059

[CR141] Ooi MF, Foo HL, Loh TC, Mohamad R, Rahim RA, Ariff A (2021) A refined medium to enhance the antimicrobial activity of postbiotic produced by *Lactiplantibacillus**plantarum* RS5. Sci Rep 11(1):7617. 10.1038/s41598-021-87081-6 33828119 10.1038/s41598-021-87081-6 PMC8027010

[CR142] Osman A, El-Gazzar N, Almanaa TN, El-Hadary A, Sitohy M (2021) Lipolytic postbiotic from *Lactobacillus**paracasei* manages metabolic syndrome in albino wistar rats. Molecules 26(2):472. 10.3390/molecules26020472 33477482 10.3390/molecules26020472 PMC7831067

[CR143] Păcularu-Burada B, Bahrim GE (2021). Extraction and antioxidant activity assessment of postbiotic exopolysaccharides produced by selected lactic acid bacteria. Innov. Roma. Food Biotechnol (20). https://www.gup.ugal.ro/ugaljournals/index.php/IFRB/article/view/4547

[CR144] Pan Y, Ma Q, Yu G, Cao Y, Jiang N, Zhang Y, Zhang S, Liu X (2024) Fermented *Lactobacillus mucilaginosus* JYLF-315 for improving skin aging, and metagen preparation and application thereof. Patent no: CN117736942B

[CR145] Park OJ, Kim J, Yang J, Yun CH, Han SH (2017) Muramyl dipeptide, a shared structural motif of peptidoglycans, is a novel inducer of bone formation through induction of Runx2. J Bone Miner Res 32(7):1455–1468. 10.1002/jbmr.3137 28337794 10.1002/jbmr.3137

[CR146] Patil S, Sawant S, Hauff K, Hampp G (2019) Validated postbiotic screening confirms presence of physiologically active metabolites, such as short-chain fatty acids, amino acids and vitamins in Hylak® Forte. Probiot Antimicrob Prot 11:1124–1131. 10.1007/s12602-018-9497-5 10.1007/s12602-018-9497-5 30560425

[CR147] Paul Beulah BF, Rajasekar T (2023) Preparation of postbiotics from Bacillus. In: Dharumadurai D (ed) Postbiotics (methods and protocols in food science). Humana, New York, pp 75–79. 10.1007/978-1-0716-3421-9_11

[CR148] Peluzio Md CG, Martinez JA, Milagro FI (2021) Postbiotics: metabolites and mechanisms involved in microbiota-host interactions. Trends Food Sci Technol 108:11–26. 10.1016/j.tifs.2020.12.004

[CR149] Peng C, David PR (2023) A postbiotic S515 production formula with lipid-lowering and metabolism-regulating functions and its application. Patent no: LU502431B1

[CR150] Poeta M, Cioffi V, Tarallo A, Damiano C, Lo Vecchio A, Bruzzese E, Parenti G, Guarino A (2023) Postbiotic Preparation of *Lacticaseibacillus**rhamnosus* GG against diarrhea and oxidative stress induced by spike protein of SARS-CoV-2 in human enterocytes. Antioxidants 12(10):1878. 10.3390/antiox12101878 37891957 10.3390/antiox12101878 PMC10604595

[CR151] Popova M, Molimard P, Courau S, Crociani J, Dufour C, Le Vacon F, Carton T (2012) Beneficial effects of probiotics in upper respiratory tract infections and their mechanical actions to antagonize pathogens. J Appl Microbiol 113(6):1305–1318. 10.1111/j.1365-2672.2012.05394.x 22788970 10.1111/j.1365-2672.2012.05394.x PMC7166318

[CR152] Pradhan D, Gulati G, Avadhani R, Rashmi H, Soumya K, Kumari A, Gupta A, Dwivedi D, Kaushik JK, Grover S (2023) Postbiotic lipoteichoic acid of probiotic *Lactobacillus**origin* ameliorates inflammation in HT-29 cells and colitis mice. Int J Biol Macromol 236:123962. 10.1016/j.ijbiomac.2023.123962 36907160 10.1016/j.ijbiomac.2023.123962

[CR153] Prajapati N, Patel J, Singh S, Yadav VK, Joshi C, Patani A, Prajapati D, Sahoo DK, Patel A (2023) Postbiotic production: Harnessing the power of microbial metabolites for health applications. Front Microbiol 14:1306192. 10.3389/fmicb.2023.1306192 38169918 10.3389/fmicb.2023.1306192 PMC10758465

[CR154] Precision Business Insights (2023) Probiotics, Prebiotics, and Postbiotics Market: By Product Type (Probiotics and Prebiotics, Probiotics, Postbiotics), By Nutrition Area (Gastrointestinal Health, Immune Health, Oral Health, Skin Health, Others) By Application Areas (Foods and Beverages, Dietary Supplements, Infant Nutrition, Others), and Geography. https://www.precisionbusinessinsights.com/market-reports/probiotics-prebiotics-and-postbiotics market#:~:text=Probiotics%2C%20Prebiotics%2C%20and%20Postbiotics%20Market%20size%20was%20valued%20at%20USD,bacteria%20in%20the%20human%20gut . Accessed 15 Sept 2024

[CR155] Qiao S, Lv C, Zhang X, Lv X, Yang D, Zhao J (2024) Antioxidant and anti-stress properties of postbiotics produced by *Lysinibacillus**macroides* G117. Comp Immunol Rep 6:200143. 10.1016/j.cirep.2024.200143

[CR156] Rad A, Aghebati-Maleki L, Kafil H, Gilani N, Abbasi A, Khani N (2021a) Postbiotics, as dynamic biomolecules, and their promising role in promoting food safety. Biointerface Res Appl Chem 11(6):14529–14544. 10.33263/BRIAC116.1452914544

[CR157] Rad AH, Aghebati-Maleki L, Kafil HS, Abbasi A (2021b) Molecular mechanisms of postbiotics in colorectal cancer prevention and treatment. Crit Rev Food Sci Nutr 61(11):1787–1803. 10.1080/10408398.2020.1765310 32410512 10.1080/10408398.2020.1765310

[CR158] Rad AH, Hosseini S, Pourjafar H (2022) Postbiotics as dynamic biological molecules for antimicrobial activity: a mini review. Biointerface Res. Appl. Chem 12(5):6543–6556. 10.33263/BRIAC125.65436556

[CR159] Ragavan ML, Hemalatha S (2024) The functional roles of short chain fatty acids as postbiotics in human gut: future perspectives. Food Sci Biotechnol 33(2):275–285. 10.1007/s10068-023-01414-x 38222911 10.1007/s10068-023-01414-x PMC10786766

[CR160] Rahman MS, Lee Y, Park DS, Kim YS (2023) *Bifidobacterium bifidum* DS0908 and *Bifidobacterium**longum* DS0950 culture-supernatants ameliorate obesity-related characteristics in mice with high-fat diet-induced obesity. J Microbiol Biotechnol 33(1):96. 10.4014/jmb.2210.10046 36457182 10.4014/jmb.2210.10046 PMC9899789

[CR161] Rezaie N, Bagheri-Amiri F, Aghamohammad S, Khatami S, Talebi M, Sohrabi A, Pourshafie MR, Rohani M (2025) The therapeutic effect of antioxidant postbiotic cocktail in colitis mice: a promising approach to alleviate oxidative stress in two high-fat and normal-diet feeding mice. J Agricult Food Res 19:101547. 10.1016/j.jafr.2024.101547

[CR162] Rezaie N, Aghamohammad S, Haj Agha Gholizadeh Khiavi E, Khatami S, Sohrabi A, Rohani M (2024) The comparative anti-oxidant and anti-inflammatory efficacy of postbiotics and probiotics through Nrf-2 and NF-kB pathways in DSS-induced colitis model. Sci. Rep 14(1): 11560. 10.1038/s41598-024-62441-0 10.1038/s41598-024-62441-0 PMC11109304 38773299

[CR163] Ríus AG, Kaufman JD, Li MM, Hanigan MD, Ipharraguerre IR (2022) Physiological responses of Holstein calves to heat stress and dietary supplementation with a postbiotic from *Aspergillus oryzae*. Sci Rep 12(1):1587. 10.1038/s41598-022-05505-3 35091685 10.1038/s41598-022-05505-3 PMC8799720

[CR164] Rivera-Jiménez J, Berraquero-García C, Pérez-Gálvez R, García-Moreno PJ, Espejo-Carpio FJ, Guadix A, Guadix EM (2022) Peptides and protein hydrolysates exhibiting anti-inflammatory activity: Sources, structural features and modulation mechanisms. Food Funct 13(24):12510–12540. 10.1039/D2FO02223K 36420754 10.1039/d2fo02223k

[CR165] Robles-Vera I, Toral M, Romero M, Jiménez R, Sánchez M, Pérez-Vizcaíno F, JJC D (2017) Antihypertensive effects of probiotics. Curr Hypertens Rep 19(4):26. 10.1007/s11906-017-0723-4 28315049 10.1007/s11906-017-0723-4

[CR166] Rocchetti MT, Russo P, De Simone N, Capozzi V, Spano G, Fiocco D (2024) Immunomodulatory activity on human macrophages by cell-free supernatants to explore the probiotic and postbiotic potential of *Lactiplantibacillus plantarum* strains of plant origin. Probiot Antimicrob Prot 16(3):911–926. 10.1007/s12602-023-10084-4 10.1007/s12602-023-10084-4 PMC11126452 37202651

[CR167] Roux E, Nicolas A, Valence F, Siekaniec G, Chuat V, Nicolas J, Le Loir Y, Guédon E (2022) The genomic basis of the *Streptococcus**thermophilus* health-promoting properties. BMC Genom 23(1):210. 10.1186/s12864-022-08459-y 10.1186/s12864-022-08459-y PMC8925076 35291951

[CR168] Rozhkova IV, Yurova EA, Leonova VA (2023) evaluation of the amino acid composition and content of organic acids of complex postbiotic substances obtained on the basis of metabolites of probiotic bacteria *Lacticaseibacillus**paracasei* ABK and *Lactobacillus**helveticus* H9. Fermentation 9(5):460. 10.3390/fermentation9050460

[CR169] Rui W, Zhong S, Li X, Tang X, Wang L, Yang J (2024) Evaluating the role of postbiotics in the modulation of human oral microbiota: a randomized controlled clinical trial. Probiotics Antimicrob Prot. 10.1007/s12602-024-10238-y 10.1007/s12602-024-10238-y 38502383

[CR170] Sabahi S, Homayouni Rad A, Aghebati-Maleki L, Sangtarash N, Ozma MA, Karimi A, Hosseini H, Abbasi A (2023) Postbiotics as the new frontier in food and pharmaceutical research. Crit Rev Food Sci Nutr 63(26):8375–8402. 10.1080/10408398.2022.2056727 35348016 10.1080/10408398.2022.2056727

[CR171] Sadeghi A, Ebrahimi M, Kharazmi MS, Jafari SM (2023) Effects of microbial-derived biotics (meta/pharma/post-biotics) on the modulation of gut microbiome and metabolome; general aspects and emerging trends. Food Chem 411:135478. 10.1016/j.foodchem.2023.135478 36696721 10.1016/j.foodchem.2023.135478

[CR172] Saeed A, Yasmin A, Baig M, Khan K, Heyat MBB, Akhtar F, Batool Z, Kazmi A, Wahab A (2023) Shahid M (2023) Isolation and characterization of *Lactobacillus**crispatus, Lactococcus**lactis,* and *Carnobacterium**divergens* as potential probiotic bacteria from fermented black and green olives (Olea europaea): an exploratory study. BioMed Res Int 1:8726320. 10.1155/2023/8726320 10.1155/2023/8726320 PMC10156456 37152587

[CR173] Salminen S, Collado MC, Endo A, Hill C, Lebeer S, Quigley EM, Sanders ME, Shamir R, Swann JR, Szajewska H (2021) The International Scientific Association of Probiotics and Prebiotics (ISAPP) consensus statement on the definition and scope of postbiotics. Nat Rev Gastroenterol Hepatol 18(9):649–667. 10.1038/s41575-021-00440-6 33948025 10.1038/s41575-021-00440-6 PMC8387231

[CR174] Salva S, Tiscornia I, Gutiérrez F, Alvarez S, Bollati-Fogolín M (2021) *Lactobacillus rhamnosus* postbiotic-induced immunomodulation as safer alternative to the use of live bacteria. Cytokine 146:155631. 10.1016/j.cyto.2021.155631 34252871 10.1016/j.cyto.2021.155631

[CR175] Santana GB, Quelemes PV, da Silva Neta ER, de Lima SG, Vale GC (2024) Chemical characterization and effect of a *Lactobacilli*-postbiotic on *Streptococcus mutans* biofilm in vitro. Microorganisms 12(5):843. 10.3390/microorganisms12050843 38792672 10.3390/microorganisms12050843 PMC11124186

[CR176] Sato N, Garcia-Castillo V, Yuzawa M, Islam MA, Albarracin L, Tomokiyo M, Ikeda-Ohtsubo W, Garcia-Cancino A, Takahashi H, Villena J, Kitazawa H (2020) Immunobiotic Lactobacillus jensenii TL2937 alleviates dextran sodium sulfate-induced colitis by differentially modulating the transcriptomic response of intestinal epithelial cells. Front Immunol 11:2174 33042131 10.3389/fimmu.2020.02174 PMC7527445

[CR177] Segers K, Declerck S, Mangelings D, Heyden YV, Eeckhaut AV (2019) Analytical techniques for metabolomic studies: a review. Bioanalysis 11(24):2297–2318. 10.4155/bio-2019-0014 31845604 10.4155/bio-2019-0014

[CR178] Seidler Y, Rimbach G, Lüersen K, Vinderola G, Ipharraguerre IR (2024) The postbiotic potential of A*spergillus oryzae*–a narrative review. Fronti. Microbiol: 151452725. 10.3389/fmicb.2024.1452725 10.3389/fmicb.2024.1452725 PMC11538067 39507340

[CR179] Semenov AV (2021) Peptidoglycan of bacterial cell wall affects competitive properties of microorganisms. Bull Exp Biol Med 172(2):164–168. 10.1007/s10517-021-05356-4 34855091 10.1007/s10517-021-05356-4

[CR180] Sergey YD, Maxim AZ, Račkauskas R (2023) Plant-based probiotic composition and method for its obtaining. Patent no: RU2790676C1

[CR181] Sevin S, Karaca B, Haliscelik O, Kibar H, OmerOglou E, Kiran F (2021) Postbiotics secreted by Lactobacillus sakei EIR/CM-1 isolated from cow milk microbiota, display antibacterial and antibiofilm activity against ruminant mastitis-causing pathogens. Ital J Animal Sci 20(1):1302–1316. 10.1080/1828051X.2021.1958077

[CR182] Shafipour Yordshahi A, Moradi M, Tajik H, Molaei R (2020) Design and preparation of antimicrobial meat wrapping nanopaper with bacterial cellulose and postbiotics of lactic acid bacteria. Int J Food Microbiol 321:108561. 10.1016/j.ijfoodmicro.2020.108561 32078868 10.1016/j.ijfoodmicro.2020.108561

[CR183] Shawn T (2023) Nutritional supplements and methods of nutritional supplementation affecting weight loss. Patent no: US20230217979A1

[CR184] Sheri S, Tye J, Kovarik JE (2024) Method and system for modulating an individual's skin microbiome. Patent no: US20240024378A1

[CR185] Shi J, Wang Q, Ruan G, Chen Y, Zhao M, Shi D, Pan B, Xu Z, Zhang T, Wang F (2023) Efficacy of probiotics against dental caries in children: a systematic review and meta-analysis. Crit Rev Food Sci Nutr 63(29):9977–9994. 10.1080/10408398.2022.2077693 35607893 10.1080/10408398.2022.2077693

[CR186] Shi L, Liu T, Song W, Wang Y (2024) Metagen composite fermentation liquor, preparation method and application thereof. Patent no: CN117180318A

[CR187] Shin HH, Kim JH, Jung YJ, Kwak MS, Sung MH, Imm JY (2024) Postbiotic potential of *Bacillus velezensis* KMU01 cell-free supernatant for the alleviation of obesity in mice. Heliyon 10(5). 10.1016/j.heliyon.2024.e25263 10.1016/j.heliyon.2024.e25263 PMC10943329 38495172

[CR188] Shiraishi T, Yokota S, Fukiya,S, Yokota A (2016) Structural diversity and biological significance of lipoteichoic acid in Gram-positive bacteria: focusing on beneficial probiotic lactic acid bacteria. Biosci. Microbio. Food Health 35(4):147–161. 10.12938/bmfh.2016-006 10.12938/bmfh.2016-006 PMC5107633 27867802

[CR189] Siciliano RA, Reale A, Mazzeo MF, Morandi S, Silvetti T, Brasca M (2021) Paraprobiotics: a new perspective for functional foods and nutraceuticals. Nutrients 13(4):1225. 10.3390/nu13041225 33917707 10.3390/nu13041225 PMC8068161

[CR190] Sivinski SE, Meier KE, Mamedova LK, Saylor BA, Shaffer JE, Sauls-Hiesterman JA, Yoon I, Bradford BJ (2022) Effect of Saccharomyces cerevisiae fermentation product on oxidative status, inflammation, and immune response in transition dairy cattle. J Dairy Sci 105(11):8850–8865. 10.3168/jds.2022-21998 36153156 10.3168/jds.2022-21998

[CR191] Song B, Zhong Y, Zheng C, Li F, Duan Y, Deng J (2019) Propionate alleviates high-fat diet-induced lipid dysmetabolism by modulating gut microbiota in mice. J Appl Microbiol 127(5):1546–1555. 10.1111/jam.14389 31325215 10.1111/jam.14389

[CR192] Steven KK, Naseer S (2020) Probiotics and fermentation metabolites for the prevention and treatment of disease conditions in animals. Pure Cultures 2020 Inc. Patent no: US10758577B2

[CR193] Suh MG, Shin HY, Jeong EJ, Kim G, Jeong SB, Ha EJ, Choi SY, Moon SK, Shin KS, Yu KW (2023) Identification of galacturonic acid-rich polysaccharide with intestinal immune system modulating activity via Peyer’s patch from postbiotics of *Phellinus**linteus* mycelial submerged culture. Int J Biol Macromol 234:123685. 10.1016/j.ijbiomac.2023.123685 36796554 10.1016/j.ijbiomac.2023.123685

[CR194] Sun Z, Zhao Z, Fang B, Hung W, Gao H, Zhao W, Lan H, Liu M, Zhao L, Zhang M (2023) Effect of thermal inactivation on antioxidant, anti-inflammatory activities and chemical profile of postbiotics. Foods 12(19):3579. 10.3390/foods12193579 37835233 10.3390/foods12193579 PMC10572142

[CR195] Sungsoo J, Yi SG, Jeong Y, Lee Y, Hemun L, Jae KC, Wonjong L, Janghyun J, Dongho S (2023) *Lactiplantibacillus plantarum* DCF49 new strain derived from shindari and production method of functional postbiotics using the same. Patent no: KR102497827B1

[CR196] Suyeon Y, Cheolmin B, Hyun MP, Hyerin L, Hayoung K, Ban OH, Jungwoo Y (2023) Method for lowering molecular-weight of collagen using fermentation of *Lacticaseibacillus rhamnosus* IDCC 3201 and complex comprising collagen and postbiotics. Patent no: KR102491324B1

[CR197] Svetlana AR, Shpak MA, Alexey DL, Anisimov SV, Georgy SA, Serafima NS (2024) Enzymatic method of producing lactulose-containing postbiotic product. Patent no: RU2819908C1

[CR198] Tain YL, Hou CY, Chang-Chien GP, Lin S, Tzeng HT, Lee WC, Wu KLH, Yu HR, Chan JYH, Hsu CN (2023) Reprogramming effects of postbiotic butyrate and propionate on maternal high-fructose diet-induced offspring hypertension. Nutrients 15(7):1682. 10.3390/nu15071682 37049522 10.3390/nu15071682 PMC10096847

[CR199] Tao L, Lu H, Xiong J, Zhang L, Sun W, Shan X (2024) The application and potential of postbiotics as sustainable feed additives in aquaculture. Aquaculture 592:741237. 10.1016/j.aquaculture.2024.741237

[CR200] Thorakkattu P, Khanashyam AC, Shah K, Babu KS, Mundanat AS, Deliephan A, Deokar GS, Santivarangkna C, Nirmal NP (2022) Postbiotics: current trends in food and pharmaceutical industry. Foods 11:193094 10.3390/foods11193094 PMC9564201 36230169

[CR201] Tian Q, Ye H, Zhou X, Wang J, Zhang L, Sun W, Duan C, Fan M, Zhou W, Bi C (2024) Evaluating the health risk of probiotic supplements from the perspective of antimicrobial resistance. Microbiol. Spec e00019-00024. 10.3390/foods11193094 10.1128/spectrum.00019-24 PMC11705942 39655960

[CR202] Tingirikari JMR, Sharma A, Lee HJ (2024) Ethnic foods: impact of probiotics on human health and disease treatment. J Ethnic Foods 11(1):31. 10.1186/s42779-024-00243-5

[CR203] Toca Md C, Burgos F, Tabacco O, Vinderola G (2023) Postbiotics: A new member in the biotics family. Arch. Argent. Pediatr e202310168. 10.5546/aap.2023-10168.eng 10.5546/aap.2023-10168.eng 37824476

[CR204] Tong Y, Hn G, Abbas Z, Zhang J, Wang J, Cheng Q, Peng S, Yang T, Bai T, Zhou Y (2023) Optimizing postbiotic production through solid-state fermentation with *Bacillus amyloliquefaciens* J and *Lactiplantibacillus plantarum* SN4 enhances antibacterial, antioxidant, and anti-inflammatory activities. Front Microbiol 14:1229952. 10.3389/fmicb.2023.1229952 37744928 10.3389/fmicb.2023.1229952 PMC10512978

[CR205] Toushik SH, Park JH, Kim K, Ashrafudoulla M, Ulrich MSI, Mizan MFR, Roy PK, Shim WB, Kim YM, Park SH (2022) Antibiofilm efficacy of *Leuconostoc mesenteroides* J. 27-derived postbiotic and food-grade essential oils against *Vibrio parahaemolyticus*, *Pseudomonas aeruginosa*, and *Escherichia coli* alone and in combination, and their application as a green preservative in the seafood industry. Food Res. Int 156:111163. 10.1016/j.foodres.2022.111163 10.1016/j.foodres.2022.111163 35651029

[CR206] Tsai W, Fang Y, Huang T, Chiang Y, Lin C, Chang W (2023) Heat-killed Lacticaseibacillus paracasei GMNL-653 ameliorates human scalp health by regulating scalp microbiome. BMC Microbiology 23(1):121. 10.1186/s12866-023-02870-5 37120517 10.1186/s12866-023-02870-5 PMC10148562

[CR207] Uhlig F, Warda AK, Hueston CM, Draper LA, Chauvière G, Eckhardt E, Hill C, Hyland NP (2023) Physiological bioactivity of a postbiotic consisting of heat-treated lactobacilli on mouse small intestine. J Funct Foods 108:105730. 10.1016/j.jff.2023.105730

[CR208] Vailati-Riboni M, Coleman D, Lopreiato V, Alharthi A, Bucktrout R, Abdel-Hamied E, Martinez-Cortes I, Liang Y, Trevisi E, Yoon I, Loor J (2021) Feeding a Saccharomyces cerevisiae fermentation product improves udder health and immune response to a Streptococcus uberis mastitis challenge in mid-lactation dairy cows. J Animal Sci Biotechnol 12:1–19. 10.1186/s40104-021-00560-8 10.1186/s40104-021-00560-8 PMC8028142 33827684

[CR209] Vukomanović M, Žunič V, Kunej Š, Jančar B, Jeverica S, Podlipec R, Suvorov D (2017) Nano-engineering the antimicrobial spectrum of lantibiotics: activity of nisin against gram negative bacteria. Sci Rep 7(1):4324. 10.1038/s41598-017-04670-0 28659619 10.1038/s41598-017-04670-0 PMC5489483

[CR210] Walter A, Mayer C (2019) Peptidoglycan structure, biosynthesis, and dynamics during bacterial growth. In: Cohen E, Merzendorfer H (eds) Extracellular sugar-based biopolymers matrices. biologically-inspired systems. Springer, Cham, pp 237–232. 10.1007/978-3-030-12919-4_6

[CR211] Wang L, Zhu Q, Lu A, Liu X, Zhang L, Xu C, Liu X, Li H, Yang T (2017) Sodium butyrate suppresses angiotensin II-induced hypertension by inhibition of renal (pro) renin receptor and intrarenal renin–angiotensin system. J Hypertens 35(9):1899–1908. 10.1097/HJH.0000000000001378 28509726 10.1097/HJH.0000000000001378 PMC11157961

[CR212] Wang Y, Wang Y, Lin X, Gou Z, Fan Q, Jiang S (2021) Effects of Clostridium butyricum sodium butyrate, and butyric acid glycerides on the reproductive performance, egg quality, intestinal health, and offspring performance of yellow-feathered breeder hens. Front Microbiol 12:657542. 10.3389/fmicb.2021.657542 34603221 10.3389/fmicb.2021.657542 PMC8481923

[CR213] Wegh CA, Geerlings SY, Knol J, Roeselers G, Belzer C (2019) Postbiotics and their potential applications in early life nutrition and beyond. Int J Mol Sci 20(19):4673. 10.3390/ijms20194673 31547172 10.3390/ijms20194673 PMC6801921

[CR214] Wonyoung B, Shin S, Jung WH, Taerak K (2023) Composition for preventing hair loss and promoting hair growth comprising postbiotic components from heat-treat *Limosilactobacillus fermentum* LM. 1020. Patent no: KR102578662B1

[CR215] Xie P, Guo Z, Cai Y, Ho X, Guo Y, Huang Y (2022) Composition and vaginal cleaning composition for inhibiting of vaginal pathogens and uses thereof. Patent no:TW202118498A

[CR216] Xin Y, Hu C, Li Y, Yang Z, Zhang L, Li A, Li C, Liu L, Du P (2024) Immunomodulatory potential of *Lactobacillus helveticus* KLDS 1.8701 postbiotics: By regulating the Th17/Treg balance. Food Biosci 61(1048)42. 10.1016/j.fbio.2024.104842

[CR217] Xu S, Jia X, Liu Y, Pan X, Chang J, Wei W, Lu P, Petry D, Che L, Jiang X (2023) Effects of yeast-derived postbiotic supplementation in late gestation and lactation diets on performance, milk quality, and immune function in lactating sows. J. Animal Sci 101, skad201. 10.1093/jas/skad201 10.1093/jas/skad201 PMC10294553 37330668

[CR218] Xu X, Wu J, Jin Y, Huang K, Zhang Y, Liang Z (2023) Both *Saccharomyces boulardii* and its postbiotics alleviate dextran sulfate sodium-induced colitis in mice, association with modulating inflammation and intestinal microbiota. Nutrition 15(6). 10.3390/nu15061484 10.3390/nu15061484 PMC10055518 36986214

[CR219] Xuemei H, Wu L, Haikuang W (2023) Application of postbiotics of inactivated *Lactobacillus* casei IOB-P9 in blood glucose reducing. Patent no: US20230364166A1

[CR220] Yeşilyurt N, Yılmaz B, Ağagündüz D, Capasso R (2021) Involvement of probiotics and postbiotics in the immune system modulation. Biologics 1(2):89–110

[CR221] Yilmaz N, Özogul F, Moradi M, Fadiloglu EE, Šimat V, Rocha JM (2022) Reduction of biogenic amines formation by foodborne pathogens using postbiotics in lysine-decarboxylase broth. J Biotechnol 358:118–127. 10.1016/j.jbiotec.2022.09.003 36087781 10.1016/j.jbiotec.2022.09.003

[CR222] Yoon Y, Ahn B, Min J, Lee K, Park SH, Kang H (2022) Stimulatory effects of extracellular vesicles derived from Leuconostoc holzapfelii that exists in human scalp on hair growth in human follicle dermal papilla cells. Curr Issues Mol Biol 44(2):845–866. 10.3390/cimb44020058 35723343 10.3390/cimb44020058 PMC8929027

[CR223] Youn HY, Seo KH, Kim HJ, Kim YS, Kim H (2022) Effect of postbiotics derived from kefir lactic acid bacteria-mediated bioconversion of citrus pomace extract and whey on high-fat diet-induced obesity and gut dysbiosis. Food Res Int 162:111930. 10.1016/j.foodres.2022.111930 36461189 10.1016/j.foodres.2022.111930

[CR224] Yousefvand A, Pham QH, Ho TM, Amiri S, Mäkelä-Salmi N, Saris PE (2024) *Bifidobacterium* animalis subsp. lactis BB12-derived postbiotic powders enhance antioxidant and physicochemical properties of low-fat yoghurt. Food Bioproc. Technol: 1–17. 10.1007/s11947-024-03405-0

[CR225] Zábolyová N, Lauková A, Pogány Simonová M (2024) Susceptibility to postbiotics-enterocins of methicillin-resistant *Staphylococcus aureus* strains isolated from rabbits. Vet. Res. Comm: 1–9. 10.1007/s11259-024-10323-1 10.1007/s11259-024-10323-1 PMC11147817 38324077

[CR226] Zago M, Massimiliano L, Bonvini B, Penna G, Giraffa G, Rescigno M (2021) Functional characterization and immunomodulatory properties of Lactobacillus helveticus strains isolated from Italian hard cheeses. PLoS One 16(1):e0245903. 10.1371/journal.pone.0245903 33493208 10.1371/journal.pone.0245903 PMC7833162

[CR227] Zakrzewska Z, Zawartka A, Schab M, Martyniak A, Skoczeń S, Tomasik PJ, Wędrychowicz A (2022) Prebiotics, probiotics, and postbiotics in the prevention and treatment of anemia. Microorganisms 10(7):1330. 10.3390/microorganisms10071330 35889049 10.3390/microorganisms10071330 PMC9317605

[CR228] Zarour K, Zeid AF, Mohedano ML, Prieto A, Kihal M, López P (2024) *Leuconostoc mesenteroides* and *Liquorilactobacillus mali* strains, isolated from Algerian food products, are producers of the postbiotic compounds dextran, oligosaccharides and mannitol. World J Microbiol Biotechnol 40(4):114. 10.1007/s11274-024-03913-3 38418710 10.1007/s11274-024-03913-3 PMC10901973

[CR229] Zeidan AA, Poulsen VK, Janzen T, Buldo P, Derkx PM, Øregaard G, Neves AR (2017) Polysaccharide production by lactic acid bacteria: from genes to industrial applications. FEMS Microbiol Rev 41(1):S168–S200. 10.1093/femsre/fux017 28830087 10.1093/femsre/fux017

[CR230] Zhang X, Esmail GA, Alzeer AF, Arasu MV, Vijayaraghavan P, Choi KC, Al-Dhabi NA (2020) Probiotic characteristics of *Lactobacillus* strains isolated from cheese and their antibacterial properties against gastrointestinal tract pathogens. Saudi J Biol Sci 27(12):3505–3513. 10.1016/j.sjbs.2020.10.022 33304162 10.1016/j.sjbs.2020.10.022 PMC7715019

[CR231] Zhang T, Zhang W, Feng C, Kwok LY, He Q, Sun Z (2022) Stronger gut microbiome modulatory effects by postbiotics than probiotics in a mouse colitis model. NPJ Sci Food 6(1):53. 10.1038/s41538-022-00169-9 36379940 10.1038/s41538-022-00169-9 PMC9666507

[CR232] Zhang Z, Guo Q, Wang J, Tan H, Jin X, Fan Y, Liu J, Zhao S, Zheng J, Peng N (2023) Postbiotics from *Pichia kudriavzevii* promote intestinal health performance through regulation of *Limosilactobacillus reuteri* in weaned piglets. Food Funct 14(8):3463–3474. 10.1039/D2FO03695A 36912248 10.1039/d2fo03695a

[CR233] Zheng Q, Chia SL, Saad N, Song AAL, Loh TC, Foo HL (2024) Different combinations of nitrogen and carbon sources influence the growth and postbiotic metabolite characteristics of Lactiplantibacillus plantarum strains isolated from Malaysian foods. Foods 13(19):3123. 10.3390/foods13193123 39410157 10.3390/foods13193123 PMC11475368

[CR234] Zhou L, Song W, Liu T, Yan T, He Z, He W, Lv J, Zhang S, Dai X, Yuan L (2024) Multi-omics insights into anti-colitis benefits of the synbiotic and postbiotic derived from wheat bran arabinoxylan and Limosilactobacillus reuteri. Int J Biol Macromol 278:134860. 10.1016/j.ijbiomac.2024.134860 39163956 10.1016/j.ijbiomac.2024.134860

